# Three-Dimensional-QSAR and Relative Binding Affinity Estimation of Focal Adhesion Kinase Inhibitors

**DOI:** 10.3390/molecules28031464

**Published:** 2023-02-02

**Authors:** Suparna Ghosh, Seung Joo Cho

**Affiliations:** 1 Department of Biomedical Sciences, College of Medicine, Chosun University, Gwangju 501-759, Republic of Korea; 2 Department of Cellular Molecular Medicine, College of Medicine, Chosun University, Gwangju 501-759, Republic of Korea

**Keywords:** focal adhesion kinase, 3D-QSAR, molecular dynamics, MM-PB/GBSA, free energy perturbation

## Abstract

Precise binding affinity predictions are essential for structure-based drug discovery (SBDD). Focal adhesion kinase (FAK) is a member of the tyrosine kinase protein family and is overexpressed in a variety of human malignancies. Inhibition of FAK using small molecules is a promising therapeutic option for several types of cancer. Here, we conducted computational modeling of FAK-targeting inhibitors using three-dimensional structure–activity relationship (3D-QSAR), molecular dynamics (MD), and hybrid topology-based free energy perturbation (FEP) methods. The structure–activity relationship (SAR) studies between the physicochemical descriptors and inhibitory activities of the chemical compounds were performed with reasonable statistical accuracy using CoMFA and CoMSIA. These are two well-known 3D-QSAR methods based on the principle of supervised machine learning (ML). Essential information regarding residue-specific binding interactions was determined using MD and MM-PB/GBSA methods. Finally, physics-based relative binding free energy ($\Delta \Delta G_{\mathrm{RBFE}}^{A\to B}$) terms of analogous ligands were estimated using alchemical FEP simulation. An acceptable agreement was observed between the experimental and computed relative binding free energies. Overall, the results suggested that using ML and physics-based hybrid approaches could be useful in synergy for the rational optimization of accessible lead compounds with similar scaffolds targeting the FAK receptor.

## 1. Introduction

Overexpression of the FAK receptor is known for its pivotal role in cell division, proliferation, migration, adhesion, and angiogenesis through its enzymatic activities in different types of cancer progression in humans [1]. FAK, also known as protein tyrosine kinase 2 (PTK2), comprises an N-terminal four-point-one, an ezrin, radixin, moesin (FERM) domain, a catalytic kinase domain, and a C-terminal domain [2]. The FERM domain is further divided into smaller subdomains (F1, F2, and F3), directly bound to the intercellular part of the transmembrane receptor proteins and the binding site for the growth factor receptors, C-Met, p53, and mouse double minute 2 (MDM2) proteins [3]. The highly conserved kinase domain (residue 300–650) participates in catalytic activity. The C-terminal domain comprises a focal adhesion targeting (FAT) domain and two proline-rich region (PRR) motifs. There are six tyrosine residues as phosphorylation sites (Y397, Y407, Y576, Y577, Y861, and Y925) that are located throughout the FAK receptor and have been identified as critical phosphorylation sites upon binding to signaling proteins [4,5].

ATP-competitive inhibitors targeting the kinase domain are promising therapeutic interventions for several types of cancers, and many are currently being studied in advanced clinical trials. However, throughout the lead optimization process, there was an uncertain dilemma between selectivity and efficacy, demanding more collaborative efforts using computational modeling and medicinal chemistry [6].

Because the binding of inhibitor compounds to target receptors involves contributions of entropy and enthalpy, biophysical and biochemical methods are frequently used to determine binding affinity. However, these procedures are costly, time-consuming, and limited to technical challenges. On the contrary, with the advent of CPU, GPU resources, and improved force fields, computational methods have shown dramatic improvement in determining the binding affinity between biomolecules [7,8]. Methods such as molecular docking, molecular dynamics, MM-PBSA binding free energy, umbrella sampling, free energy perturbation (FEP), and thermodynamic integration (TI) have been developed and effectively used for binding affinity assessment in kinase drug design [9].

In our current work, we conducted a molecular modeling study by taking 125 analogous compounds as FAK inhibitors, which exhibited a wide spectrum of inhibitory activities [10,11,12,13,14]. These compounds are ATP-competitive inhibitors with high structural similarity to TAE226 or TAE molecules. Compound TAE226 has been shown in previous research to effectively inhibit the development of glioma and ovarian cancer cells while also increasing survival rates in animals with glioma xenograft or ovarian tumor cells [15,16]. The compounds taken from the literature for the modeling study were expected to interact with FAK in a similar manner to TAE226 (PDB: 2JKK and 4D58) [17,18]. We developed CoMFA and CoMSIA, two well-known 3D-QSAR methods, to establish the structure–activity relationship of the compounds in the dataset. Unlike 2D-QSAR, 3D-QSAR includes quantum chemical descriptors, unique molecular scaffolds, substituent constants, surface and volume descriptors, and autocorrelation descriptors. This provides richer information and better reflects the non-bonded interaction properties between the receptor and ligands. Additionally, the key structural features of the inhibitors were graphically represented as contour polyhedrons in descriptive color schemes, which are useful for designing new chemical compounds by scaffold hopping or molecular probing. The SAR investigation study was integrated with the residue-specific binding energy profile from the MM-PB/GBSA analysis. The relative binding affinity calculation for a congeneric series of small molecules has gained popularity for lead optimization in the pharmaceutical industry and institutional laboratories over the last decade. We estimated the relative binding free energy ($\Delta \Delta G_{\mathrm{RBFE}}^{A\to B}$) terms by taking 12 compounds and then correlated them with their relative experimental binding free energy ($\Delta \Delta G_{\mathrm{EXP}}^{A\to B}$) values.

## 2. Results and Discussion

### 2.1. MD Simulation Analysis and Binding Energy Calculation

**Figure 1:**
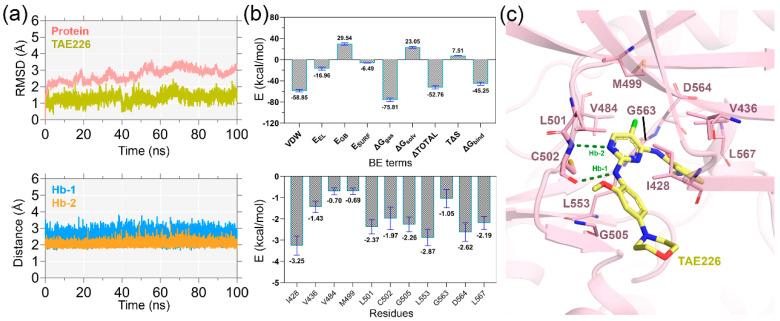
MD simulation and MM-PB/GBSA binding energy calculation. (**a**) RMSD analysis of the protein backbone and TAE226 during 100 ns of MD simulation. The distances of the two intermolecular H-bonds (Hb-1 and Hb-2) with the carbonyl and amide groups of C502 are shown during the MD run. (**b**) Binding affinity calculation and residue-specific binding energy decomposition from the MM-PB/GBSA calculation. (**c**) Residues within 4 Å distance of the TAE226 atoms, that contribute critical binding energy to the ligand, are shown in the stick representation.

The protein–ligand RMSD curves for the 100 ns MD simulation are shown in Figure 1a. Convergence was reached within the initial 5 ns interval, and thereafter, both the ligand and the protein maintained a stable plateau at the end of the production run. In the crystallographic form, TAE226 was stabilized by forming two interatomic H-bonds (Hb-1 and Hb-2) with the carbonyl and amide group of C502 with nitrogen atoms of pyrimidine and 2-methoxyaniline moieties, respectively. The chlorine atom present in the pyrimidine ring makes contact with the gatekeeper residue M499 by van der Waals interaction [17]. Additional hydrophobic interactions were observed with residues I428, A452, G505, and L553. In Figure S1, we compared the active site of the MD structure to the actual crystal structure. A critical positional displacement was noticed between the morpholine moieties of the MD and crystallographic TAE226, where no interactions with the protein were seen. The aniline and pyrimidine rings in adenine triphosphate binding pockets were almost superimposed with each other. The P-loop in the MD structure exhibited a mobility shift to the outer direction to 3.9 Å from the actual crystal structure. In MD form, the 4-aniline ring and methyl carbamoyl moiety reproduced very similar contacts with residues D564 and L567, indicating that the interaction with D564 and L567 stabilizes the DFG motif and the short α-helix.

The H-bond distances (Hb-1 and Hb-2) were measured through production simulation and were found to be between 2.7–3.5 Å, validating the overall stability of the ligand. Next, we calculated the ligand binding affinity using the MM-PB/GBSA end-state binding free energy calculation. The different binding energy (BE) terms are shown in Figure 1b and Table S1. The van der Waals (VDW) and electrostatic (E_EL_) terms each provided favorable ligand binding energies of −58.85 and −16.96 kcal/mol. The polar (E_GB_) and non-polar (E_SURF_) solvation terms were obtained as 29.54 and −6.49 kcal/mol. The ΔTOTAL and interaction entropy (−TΔS) were obtained as −52.76 and 7.51 kcal/mol, respectively. The final binding energy (${\Delta G}_{\mathrm{bind}}$) was estimated to be −45.25 kcal/mol by deducting the entropy term from ΔTOTAL. Accurate binding energy contributions from active site residues are crucial for the structure-guided inhibitor optimization process. In our study, we identified that I428, V436, V884, M499, L501, C502, G505, L553, G563, D564, and L567 were present within the boundary of 4 Å of the ligand atoms and contributed the critical binding affinity to the ligand (Table S2). This information was further co-utilized in the 3D-QSAR study to define the physicochemical impacts around certain amino acid residues.

### 2.2. Statistical Analysis of 3D-QSAR Models

**Figure 2:**
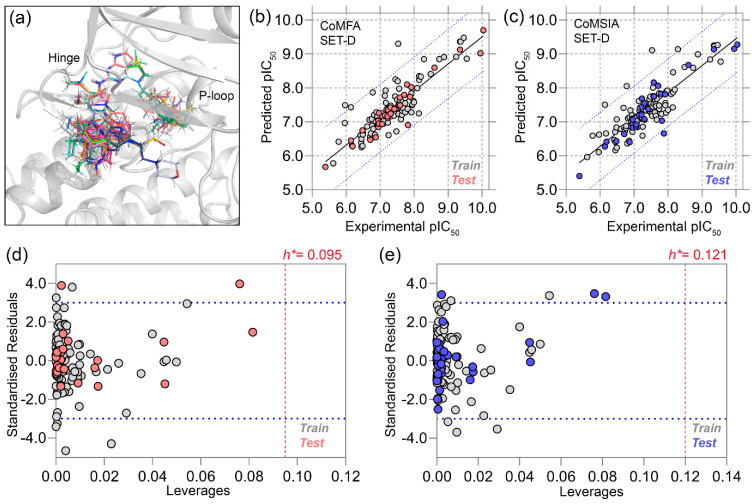
Molecular alignment of the dataset compounds, PLS plots, and applicability domain (AD) analysis. (**a**) Molecular alignment of the dataset compounds on the common chemical core by taking C36 inside the FAK binding cavity. (**b**) PLS correlation plots of the CoMFA (SET-D) study. (**c**) PLS correlation plots of the CoMSIA (SET-D) study. (**d**,**e**) Applicability domain analysis using the distance-based Williams plot using the data obtained from the CoMFA and CoMSIA models. The h* with dotted lines in red signifies the warning leverage values in both plots.

The receptor-based CoMFA and CoMSIA, two well-known 3D-QSAR models, were developed using 125 compounds. Compound C107 has non-specific bioactivity and was discarded from the dataset during model building. The 2D structures and corresponding pIC_50_ values of these compounds are listed in Table S3. Molecular alignment of the compounds was carried out by superimposing the dataset compounds over the common core of the average MD position of C36. The 3D alignment of the compounds over C36 inside the binding pocket is shown in Figure 2a. To develop a well-predictive model as well as the model’s predictive ability, we split the dataset into a training set and test set compounds by following a 3:1 ratio by employing random sampling methods according to our previous studies [19,20]. Briefly, the compounds were arranged into three mutually exclusive non-overlapping groups, i.e., high, medium, and low activity groups based on their pIC_50_ values. Following that, a random draw was performed from each group in such a way, so that the compounds had an equal chance to be selected in the test set compounds. Using this method, four different training and test sets were developed for the CoMFA study (SET-A to SET-D), as shown in Table S4.

Statistical analyses of the CoMFA models are presented in Table 1. During the model evaluation, we strictly followed the acceptance criterion for each parameter, specified in the ‘threshold value column’. The q^2^ and r^2^ values for SET-A were 0.593 and 0.839, respectively, at an ONC of 5. For SET-B, the q^2^ and r^2^ values were 0.541 and 0.666 at an ONC of 2. The q^2^ and r^2^ values of SET-C were 0.505 and 0.612 at an ONC of 2, while SET-D had q^2^ and r^2^ values of 0.633 and 0.897 at ONC of 6. Higher q^2^ and r^2^ values in combination with low χ^2^ and RMSE values were considered for the internal validation of the proposed model employing the training set compounds. SET-D had the highest q^2^ and r^2^ with satisfactory χ^2^ and RMSE values of 0.325 and 0.356, respectively, which were below the threshold constraint, and was selected as the final CoMFA model among the other datasets. In addition to the above parameters, k or k′_,_ $r_{0}^{2}$ or ${r^{\prime }}_{0}^{2}$_,_$\;\left|r_{0}^{2}-{r^{\prime }}_{0}^{2}\right|,{\;r}_{m}^{2}$ or ${r^{\prime }}_{m}^{2}$ were also computed for internal validation and were found to be in good agreement with the threshold parameters. However, QSAR models are unpredictable without external validation using test set compounds that are not included in the training set during model development. Similar to the internal validation, k or k′, $r_{0}^{2}$ or ${r^{\prime }}_{0}^{2},\;\left|r_{0}^{2}-{r^{\prime }}_{0}^{2}\right|,{\;r}_{m}^{2}$ or ${r^{\prime }}_{m}^{2}$ parameters were considered to assess the external validation of the model. However, the final selection was carried out by evaluating the predictive correlation coefficient or $r_{\mathrm{pred}}^{2}$. Overall, SET-D showed the highest $r_{\mathrm{pred}}^{2}$ value ($r_{\mathrm{pred}}^{2}=0.911,>0.6$) and was therefore considered as the final CoMFA model.

We employed the CoMSIA evaluation of SET-D since CoMSIA employed a more comprehensive set of descriptor fields, such as the hydrophobic (H), H-bond acceptor (A), and H-bond donor (D), in addition to the steric (S) and electrostatic (E) fields of CoMFA in different permutation–combination processes (Table S5). The highest q^2^ and r^2^ values were 0.656 and 0.862 at an ONC of 6, respectively. The other parameters, such as χ^2^ and RMSE, $r_{m}^{2}$ or ${r^{\prime }}_{m}^{2}$ followed the well-accepted statistical norms indicating good internal validation. In addition, we obtained an $r_{\mathrm{pred}}^{2}$ of 0.843, indicating excellent predictivity of the CoMSIA model. The actual and predicted pIC_50_ values with the residuals are listed in Table S6, and the PLS correlation plots from CoMFA and CoMSIA are shown in Figure 2b,c, respectively.

Overall, SET-D provided statistically significant CoMFA and CoMSIA models with strong internal and external validations, suggesting that both models can predict the inhibitory potential of unknown chemicals with a similar scaffold. Next, we performed the applicability domain (AD) analyses using data obtained from the 3D-QSAR study. Unlike other ML-based methods, 3D-QSAR uses the least squares algorithm to correlate the chemical descriptors and their inhibitory activity; thus, QSAR applications are limited but highly efficient for compounds with congeneric series of compounds. The applicability domain is a distance-based graphical prediction method, that determines the uncertainty in the predictability of compounds based on structural similarity. The AD analysis of CoMFA and CoMSIA using the Williams plot is depicted in Figure 2d,e in a square area of σ = ±3, in which the standardized residuals of the training and test set compounds are plotted against the leverage values. None of the compounds fell outside the warning leverage (h*), indicating the reliability and robustness of both 3D-QSAR models.

### 2.3. Contour Map Analysis

**Figure 3:**
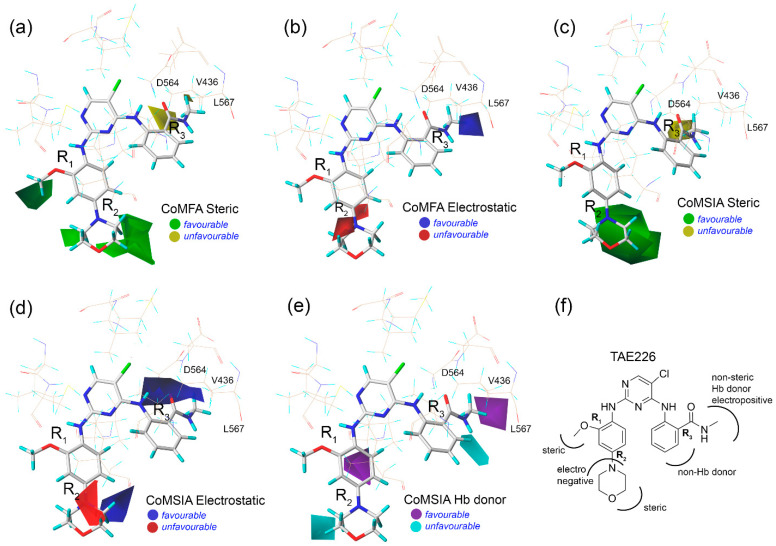
Contour map analysis and structure–activity relationship study from 3D-QSAR. (**a**) The steric contour map and (**b**) electrostatic contour map from CoMFA. (**c**–**e**) are steric, electrostatic, and H-bond (Hb) donor contour maps from CoMSIA. (**f**) Implementation of the SAR diagram from the CoMFA and CoMSIA analyses by taking TAE226 (C36) as a reference.

Following statistical validation, descriptive colored contour maps around the MD structure of C36 were generated from the 3D-QSAR study. The compounds were well aligned on the common core of the *N*-phenylpyrimidine-2-amine moiety inside the ATP pocket (Figure 3a). In the CoMFA analysis, the green and blue contours represent a favorable position for steric and electropositive substitutions, whereas the yellow and red contours did not favor those substitutions (Figure 3b,c) [21,22]. In the steric contour map, a green contour was observed near the R_1_ position of the anisole ring, and two green contours appeared around the R_2_ position of the morpholine ring, indicating that the steric substitution would be preferable for these regions. A yellow contour at the R_3_ position near residues D564, V436, and L567 indicates an unfavorable position for bulky steric substitution. Consequently, residue D564 is the part of the DFG motif that contributes −2.62 kcal/mol to ligand binding; thus, a bulky substitution at that position could have the steric hindrance effect and may lead to a decrease in overall binding affinity. Compounds C71, C72, C77, C79, C81, C82, and C84 had steric moieties adjacent to the green contours and exhibited inhibitory (pIC_50_) more than 9. In the electrostatic contour map, a blue contour near *N*-methylbenzamide and two small red contours near the morpholine ring indicate that positively charged groups would be favorable and unfavorable in that chemical space. Very similar steric and electrostatic contours appeared (Figure 3d,e) during the CoMSIA study, although an additional blue contour was present in the *ortho-* position of the six-membered rings at R_2_, overall corroborating the CoMFA contours. In the CoMSIA H-bond donor contour, two purple and two cyan contours appeared near R_2_ and R_3_, indicating the favorable and unfavorable substitutions for the H-bond donor groups, which can increase the overall inhibitory potential of C36. Figure 3f shows a SAR diagram based on the information obtained from the 3D-QSAR analysis. Residues D564, V436, and L567 were proximal (<4 Å) to the R_3_ position of *N*-methylbenzamide, and the critical binding energy decomposed to C36. Furthermore, SAR analysis revealed that non-steric, H-bond donor, and electropositive chemical groups could be advantageous substitutions at R_3_ in terms of improving inhibitory effects. Therefore, this chemical space of C36 could serve as a potential site for chemical modification to ameliorate the FAK binding affinity.

### 2.4. Relative Binding Affinity Estimation

**Figure 4:**
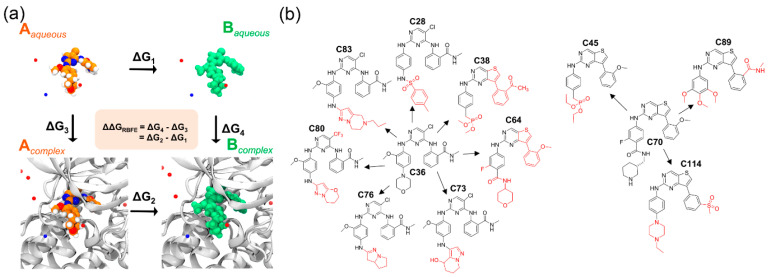
Overview of the FEP scheme and relative binding affinity estimation. (**a**) Thermodynamic pathway of ligand transformation from state-A to state-B in aqueous and complex form. The $\Delta \Delta G_{\mathrm{RBFE}}^{A\to B}$ can be deduced from the free energy changes of both states in aqueous and complex systems. (**b**) Relative binding energy calculation of the ligands through alchemical transformation. The mismatched atoms between the ligand pairs, which need to be perturbed, are shown in red.

For the relative binding estimation study, we randomly selected 12 compounds from the dataset by varying the degree of inhibitory activity. The experimental binding energy ($\Delta G_{\mathrm{EXP}}$) values were deduced from the inhibitory activities of the selected compounds. The partial charges and LJ parameters gradually changed during the alchemical transformation of the ligand from state-A to state-B within the binding pocket in the FEP simulation. These changes were made by implementing a hybrid topology from 0 to 1 in twelve different λ intermediate steps. Figure 4a shows the generalized thermodynamic cycle of the relative binding free energy derivation scheme. In the earlier studies [23,24], we used an absolute binding free energy estimate in the modeling study of kinase inhibitors and found a satisfactory correlation between the experimental and computed binding free energies. Although, the calculated binding free energies were overestimated in comparison to the corresponding experimental values. In the absolute binding affinity prediction by the FEP scheme, the entire ligand needs to be perturbed (interactions off or on) corresponding to its surroundings. This requires a large number of λ intermediate states and simulation times. In contrast, only a fraction of the chemical moiety is required to be perturbed to transition from state-A to state-B using fewer λ states. Structurally, the reported compounds consist of two types of adenine-mimicking moieties: six-membered heterocyclic rings (pyrimidine) and fused heterocyclic rings (thieno [3,2-d]pyrimidine). Therefore, it is more appropriate to select the two representative compounds as starting structures in the FEP simulation. Compounds C36 and C70 were selected as state-A, while compounds C28, C38, C45, C64, C73, C76, C80, C83, C89, and C114 were assigned as state-B. The common and mismatched atoms are shown in black and red in Figure 4b, respectively. Typically, a hybrid molecule was generated by superimposing the chemical structures of two analog ligands. In this hybrid molecule, the common part was assigned a single topology or the same topology as the first ligand. The remaining hybrid structure was assigned as a single-dual hybrid topology. During the FEP simulation, the dual topology portion was changed (including the LJ parameters, partial charges, and bonds) using the forcefields by 12 alter-λ scaling simulations (Table S7). Each alter-λ simulation was run for 1 ns in triplicate to ensure sufficient sampling while overlapping the neighboring windows. In this manner, a total of 72 ns simulations for a single alchemical transformation in complex and isolated forms were performed.

**Figure 5:**
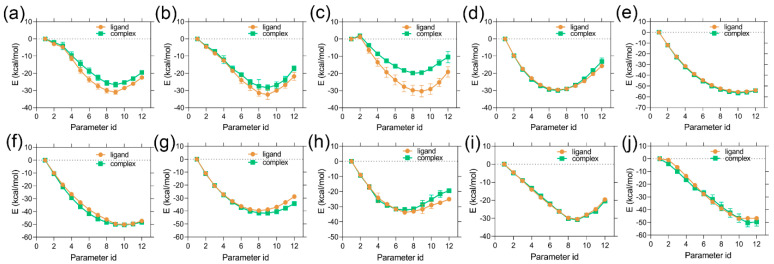
FEP energy convergence plots of (**a**) C36 → C28, (**b**) C36 → C38, (**c**) C36 → C64, (**d**) C36 → C73, (**e**) C36 → C76, (**f**) C36 → C80, (**g**) C36 → C83, (**h**) C70 → C45, (**i**) C36 → C89, and (**j**) C36 → C114 in isolated and complex form.

The results of the alchemical transformation by the FEP method are shown in Figure 5, as the free energy changes from state-A to state-B through different λ states in complex and isolated forms. BAR methods were utilized to calculate the energy differences between neighboring λ windows. In each graph, the total energy differences between the initial (λ = 0) and final (λ = 1) stages of the ligands in the complex and isolated forms correspond to $\Delta G_{\mathrm{COM}}^{A\to B}$ and $\Delta G_{\mathrm{LIG}}^{A\to B}$, respectively. From Equation (3), we derived the $\Delta \Delta G_{\mathrm{RBFE}}^{A\to B}$ from each ligand transformation, which is summarized in Table 2. The computed $\Delta \Delta G_{\mathrm{RBFE}}^{A\to B}$ of C36 → C28, C36 → C38, C36 → C73, C36 → C76, C36 → C83, C36 → C89, and C36 → C114 were found to be 2.94, 4.58, 2.64, −0.23, −0.97, −0.79, and −2.86 kcal/mol corresponding to their theoretical $\Delta \Delta G_{\mathrm{EXP}}^{A\to B}$ of 1.44, 3.00, 0.93, −1.43, −0.53, −0.60, and −1.29 kcal/mol, respectively, which is a good agreement between the experimental and computed relative binding affinity. However, the transformation of C36 → C64, C70 → C45, and C70 → C114 yielded a higher $\Delta \Delta G_{\mathrm{RBFE}}^{A\to B}$ approximation than the $\Delta \Delta G_{\mathrm{EXP}}^{A\to B}$ values. In this case, we anticipated that increasing the number of iterations and λ sampling would reduce the mean statistical approximation. We determined the Pearson’s correlation coefficient using the computed values and their respective experimental values in Figure S2. A Pearson’s R ($R_{\mathrm{RBFE}}$) was obtained as 0.82 and an R^2^ of 0.68, indicating the reasonable performance of the physics-based binding affinity calculation. In addition, the correlation statistics can be expressed in a linear equation form:

(1) $$\Delta \Delta G_{\mathrm{EXP}}^{A\to B}=0.3345\times \Delta \Delta G_{\mathrm{RBFE}}^{A\to B}\;-0.4229$$

The above equation could be useful for FEP-based SAR investigation of TAE226/C36 analogs as well as the prediction of $\Delta \Delta G_{\mathrm{EXP}}^{A\to B}$ values with reasonable accuracy.

## 3. Methodology

### 3.1. Structure Preparation

The bis-anilino pyrimidine (BI9)/TAE226-bound FAK complex with a resolution of 1.95 Å was retrieved from the RCSB PDB database (PDB ID 4D58). The crystallographic water molecules and ions were removed. The missing atoms, side chains, and loops were modeled using the web version of MODELLER in Chimera-1.15, according to our previous studies [25,26]. SYBYL was used to perform the necessary naming and atom index adjustment of the TAE226 molecule so that it was compatible with the AMBER forcefield during the all-atom MD simulation.

### 3.2. MD Simulation and Binding Energy Calculation

The all-atom MD simulation of the protein–ligand complex was conducted by GROMACS version: 2019.5 [27], using the Amber ff03 force field, according to earlier studies [28,29]. TAE226 was parameterized using ACEPYPE [30], where the atom types were assigned as GAFF types and the AM1-BCC partial charge model. The complex was placed in the center of a cubic periodic box and solvated using the TIP3P water model. The minimum thickness of the water wall was maintained at 10 Å from the protein atoms. The solvated complex was neutralized and then ionized using an adequate amount of Na^+^ and Cl^-^ ions to bring the final salt concentration to 150 mM. Next, the system was energy minimized for 10,000 steps, followed by 200 ps of NVT, 400 ps of NPT, and 100 ns of MD production simulations. In the NVT and NPT simulations, a modified Berendsen thermostat and barostat were used to achieve the 300 K temperature and 1 bar of pressure, respectively. The backbone of the protein and the heavy atoms of the ligands are restrained during the NVT and NPT ensembles, while they were omitted during the production run. The built-in ‘gmx rms’ function was used to calculate the RMSD of the protein and the ligand, respectively. More methodological details can be found in our previous study [28]. The MM-PB/GBSA binding free energy (${\Delta G}_{\mathrm{bind}}$), as well as the entropy term (TΔS) between the protein and ligand, was computed using the gmx_MMPBSA [31] package, as described in the previous study [29]. The binding energy (${\Delta G}_{\mathrm{bind}}$) obtained from the MM-PB/GBSA calculation can be expressed as follows:

(2) $${\Delta G}_{\mathrm{bind}}={\Delta G}_{\mathrm{COM}}-\left({\Delta G}_{\mathrm{PROT}}+{\Delta G}_{\mathrm{LIG}}\right)$$

where ${\Delta G}_{\mathrm{COM}}$, ${\Delta G}_{\mathrm{PROT}}$, and ${\Delta G}_{\mathrm{LIG}}$ represent the total free energy of the complex, protein, and ligand in the solvent, respectively.

### 3.3. Dataset Preparation and Molecular Modeling

A total of 125 compounds were acquired from the previously published literature and their inhibitory activity (IC_50_) values were translated to-logIC_50_ (pIC_50_). Compound C36 is already available as bis-anilino pyrimidine (BI9) or TAE226 in a high-resolution co-crystallized form bound with FAK (PDB ID 4D58). Moreover, we employed the MD ensemble to obtain a fully equilibrated protein–ligand structure complex. Therefore, the last frame of C36 from the MD trajectory was considered to be a biological 3D conformer and represented the template molecules of the dataset. Based on the template molecule, the rest of the compounds were sketched, minimized, and assigned Gasteiger–Hückel partial charges in SYBYL, as described here [32].

### 3.4. Development of 3D-QSAR Models

The compounds were aligned to the common chemical core using the template molecule (C36) as a reference. The compounds were then classified into low, medium, and high activity classes, and the test set compounds were chosen at random from each class to achieve a final training vs. test set ratio of 3:1. CoMFA and CoMSIA were used to develop 3D-QSAR models. In both methods, the chemical descriptor fields were calculated in a 3D cubic box with a grid spacing of 1 Å. At each grid intersection, a hybridized sp^3^ carbon atom with a +1 charge was assigned to compute the steric (S) and electrostatic (E) fields. In CoMSIA, an additional three fields, namely, hydrophobic (H), H-bond acceptor (A), and H-bond donor (D), with a Gaussian function. The partial least squares (PLS) method was used to assess the statistical correlation between the chemical descriptors and inhibitory activities in the CoMFA and CoMSIA models. Leave-one-out and fit procedures were applied to obtain the squared correlation coefficient of the cross-validation (q^2^) and the squared correlation coefficient (r^2^) of the fit by taking the training set compounds, followed by predicting the pIC_50_ of every compound in the dataset including the test set compounds. The external validation or predictivity of the QSAR models was determined by calculating the predictive squared correlation coefficient or r^2^_pred_ values. Additional parameters such as k or k′, $r_{0}^{2}$ or ${r^{\prime }}_{0}^{2}$, $\left|r_{0}^{2}-{r^{\prime }}_{0}^{2}\right|$, $r_{m}^{2}$ or ${r^{\prime }}_{m}^{2}$, $Q_{F1,}^{2}$ $Q_{F2}^{2}$, $Q_{F3}^{2}$, and $Q_{\mathrm{ccc}}^{2}$ were also considered for the reliability of the model according to these studies [33,34]. The applicability domain (AD) of the developed CoMFA and CoMSIA models was evaluated using a distance-based Williams plot according to this study [35]. The field distributions of the descriptors were vividly represented as descriptive colored contours, suggesting favorable and unfavorable chemical substitutions that could increase the inhibitory potency of the lead compounds.

### 3.5. Relative Binding Energy Calculation

According to this study [36], the relative binding free energy was computed by GENESIS 1.7.1 [37] using a hybrid topology approach with a CHARMM36 [38] force field. Briefly, C36 and C70 were selected as state-A molecules. On the other hand, compounds C28, C38, C45, C64, C73, C76, C80, C83, C89, and C114 were selected as state-B molecules. These compounds were randomly selected from the dataset based on their variable inhibitory activities. The hybrid ligand’s structure, topology, parameters, and input files were generated using CHARMM-GUI [39]. The maximum common substructure (MCS) was applied for overlapping ligands to determine the minimal perturbated atoms between the paired ligands. If such a state-A to state-B mutation is not feasible for a certain ligand, the CGenFFv1.x algorithm discards it automatically and is not considered further. For the simulation setup, two end-state systems were generated for each paired ligand, i.e., the ligand in the solvent and the ligand in the complex. The systems were neutralized and ionized with 0.15 M NaCl counterion. Next, minimization, NVT, and NPT simulations were performed to remove the bad contacts, gradually increasing the temperature from 0.1 K to 300 K and the pressure to 1 bar with the application of the restraint. Following that, a long 10 ns second NPT simulation was performed without position restraint. Thereafter, the λ-exchange FEP simulations were performed. Twelve λ windows were used to sequentially transform the interactions from state-A to state-B with the surroundings, in which six coupling parameters were used. Finally, the free energy differences were estimated using the Bennett acceptance ratio (BAR) method. The relative binding free energy ($\Delta \Delta G_{\mathrm{RBFE}}^{A\to B}$) between the paired ligands was calculated as the following:

(3) $$\Delta \Delta G_{\mathrm{RBFE}}^{A\to B}=\Delta G_{\mathrm{COM}}^{A\to B}-\Delta G_{\mathrm{LIG}}^{A\to B}$$

where $\Delta G_{\mathrm{COM}}^{A\to B}$ and $\Delta G_{\mathrm{LIG}}^{A\to B}$ represent the free energy changes upon the transformation of state-A to state-B in the complex and isolated in solution, respectively.

## 4. Conclusions

In this study, we employed a hybrid modeling approach based on ML and physics to study the structure–activity relationship and the binding mechanism of *N*-phenylpyrimidine-2-amine-based FAK inhibitors. As FAK is one of the key regulators of growth factor receptor signaling, its overexpression and concomitant drug resistance pose a major challenge for chemists. In the molecular simulation, H-bond analysis and MM-PB/GBSA binding energy calculations were employed to assess the ligand stability, binding affinity, and per-residue binding energy decomposition of the crystal ligand. Residues such as I428, V436, V484, M499, L501, C502, G505, L553, G563, D564, and L567 were identified as critical BE-contributing residues to ligand binding. Following that, the statistically reasonable CoMFA (q^2^ = 0.633, r^2^ = 0.897) and CoMSIA (q^2^ = 0.656, r^2^ = 0.862) models were developed, both of which showed excellent predictive capability ($r_{\mathrm{pred}}^{2}>0.6$). Descriptive colored contour maps surrounding compound C36 illustrated that chemical substitutions along these contours would more likely increase inhibitory activity. This information can be further co-utilized with the residue-specific binding energy profile to aid in molecular probing and ligand design. Finally, we applied the alchemical FEP simulation by taking 12 different ligands to estimate their relative binding affinity. An acceptable agreement was obtained between the experimental relative binding energy and the computed relative binding energy. The ML-based approach is fast and computationally economical, allowing hundreds to thousands of chemical compounds to be evaluated for biophysical and biochemical properties in early-stage drug discovery. However, the dynamic behaviors and the presence of receptor molecules are often neglected. Additionally, in certain circumstances, the ML tends to overfit the data points, thus reducing the transferability of the proposed model. In contrast, when experimental data for a few known ligands are available, physics-based methods may reasonably predict the binding affinity. However, the availability of the high-resolution crystal structure and computational resources is a major challenge for achieving an acceptable benchmark in the physics-based model. Thus, the hybrid modeling techniques employed here appear to be complementary to each other and accurate within their domain of applicability. The overall study could be beneficial for further lead optimization in medicinal chemistry research or could be applicable to other systems.

## Figures and Tables

**Table 1:** Statistics of the CoMFA and CoMSIA models.

Statistical Parameters	CoMFA				CoMSIA (SED) SET-D	Threshold Values	Statistical Parameters	CoMFA				CoMSIASET-D	Threshold Values
	SET-A	SET-B	SET-C	SET-D				SET-A	SET-B	SET-C	SET-D		
q^2^	0.593	0.541	0.505	0.633	0.656	>0.5	k_Test_	0.994	0.979	1.009	1.007	1.011	0.85 ≤ k or k′ ≤ 1.15
ONC	5	2	2	6	6		k′_Test_	1.002	1.015	0.985	0.991	0.985	
SEP	0.559	0.554	0.612	0.521	0.510		r^2^_Test_	0.578	0.422	0.767	0.922	0.850	
r^2^	0.839	0.666	0.643	0.897	0.862	>0.6	$r_{0}^{2}$ _Test_	0.494	0.377	0.735	0.915	0.854	≈r^2^
SEE	0.352	0.473	0.277	0.277	0.323	<<1	${r^{\prime }}_{0}^{2}$ _Test_	0.540	0.240	0.417	0.886	0.816	
F-value	91.487	90.592	81.911	125.822	89.719		$\left|r_{0}^{2}-{r^{\prime }}_{0}^{2}\right|$ _Test_	0.046	0.137	0.317	0.028	0.037	<0.3
BS-r^2^	0.895	0.712	0.699	0.934	0.940		$\frac{r^{2}-r_{0}^{2}}{r^{2}}$ _Test_	0.144	0.104	0.317	0.007	−0.003	<0.1
BS-SD	0.025	0.051	0.050	0.017	0.016		$\frac{r^{2}-{r^{\prime }}_{0}^{2}}{r^{2}}$ _Test_	0.064	0.430	0.041	0.038	0.039	
χ^2^	0.285	0.537	0.507	0.387	0.325	<1.0	$r_{m}^{2}$ _Test_	0.410	0.333	0.630	0.846	N/A	$r_{m}^{2}$$\;\mathrm{or}\;{r^{\prime }}_{m}^{2}$ > 0.5
RMSE	0.333	0.437	0.430	0.382	0.356	<0.5	${r^{\prime }}_{m}^{2}$ _Test_	0.466	0.242	0.313	0.748	0.694	
MAE	0.001	0.001	0.001	0.001	0.001	≈0	$r_{\mathrm{pred}}^{2}$	0.495	0.361	0.724	0.911	0.843	>0.6
RSS	14.275	24.554	23.748	15.253	16.28		$Q_{F1}^{2}$	0.495	0.361	0.724	0.911	0.843	
k_Train_	0.996	1.003	0.998	0.991	0.997	0.85 ≤ k or k′ ≤ 1.15	$Q_{F2}^{2}$	0.493	0.353	0.723	0.910	0.842	
k′_Train_	1.000	0.991	0.996	1.005	0.999		$Q_{F3}^{2}$	0.495	0.361	0.724	0.911	0.843	
$r_{0}^{2}$ _Train_	0.814	0.665	0.597	0.667	0.718	≈r^2^	$Q_{\mathrm{ccc}}^{2}$	0.759	0.655	0.811	0.950	0.916	
${r^{\prime }}_{0}^{2}$ _Train_	0.785	0.396	0.467	0.635	0.662		S (%)	47.1	47.0	46.9	39.4	18.7	
$\left|r_{0}^{2}-{r^{\prime }}_{0}^{2}\right|$ _Train_	0.028	0.269	0.129	0.041	0.055	<0.3	E (%)	52.9	53.0	53.1	60.6	46.1	
$\frac{r^{2}-r_{0}^{2}}{r^{2}}$ _Train_	0.029	2.53 × 10^−5^	0.071	0.245	0.167	<0.1	H (%)						
$\frac{r^{2}-{r^{\prime }}_{0}^{2}}{r^{2}}$ _Train_	0.063	0.404	0.273	0.291	0.231		A (%)						
$r_{m}^{2}$ _Train_	0.706	0.663	0.505	0.476	0.534	$r_{m}^{2}$$\;\mathrm{or}\;{r^{\prime }}_{m}^{2}$ > 0.5	D (%)					35.2	
${r^{\prime }}_{m}^{2}$ _Train_	0.644	0.320	0.373	0.438	0.477								

q^2^: squared cross-validated correlation coefficient; ONC: optimal number of components; SEP: standard error of prediction; r^2^: squared correlation coefficient; SEE: standard error of estimation; F-value: F-test value; BS-r^2^: bootstrapping squared correlation coefficient; χ^2^: chi-square value; RMSE: root mean square error; MAE: mean absolute error; k: slope of the predicted vs. observed activity at zero intercepts; k′: slope of the observed vs. predicted activity at zero intercepts; $r_{0}^{2}$: squared correlation coefficient between predicted and observed activity; ${r^{\prime }}_{0}^{2}$: squared correlation coefficient between predicted and observed activity; $r_{m}^{2}$ or ${r^{\prime }}_{m}^{2}$: $r_{m}^{2}$, ${r^{\prime }}_{m}^{2}$ matrix; $r_{\mathrm{pred}}^{2}$: predictive correlation coefficient; $Q_{F1}^{2}$, $Q_{F2}^{2}$, $Q_{F3}^{2}$, and $Q_{\mathrm{ccc}}^{2}$: $Q_{F1}^{2}$, $Q_{F2}^{2}$, $Q_{F3}^{2}$ and $Q_{\mathrm{ccc}}^{2}$ statistical measures/parameters. S: steric; E: electrostatic; H: hydrophobic; A: H-bond acceptor; D: H-bond donor.

**Table 2:** Energy terms of alchemical binding energy transformation from state-A to state-B.

$\mathrm{State}-A\;(\Delta G_{\mathrm{EXP}})$	$\mathrm{State}-B\;(\Delta G_{\mathrm{EXP}})$	$\Delta \Delta G_{\mathrm{EXP}}^{A\to B}$	$\Delta G_{\mathrm{COM}}\;\pm \;\mathrm{SD}$	$\Delta G_{\mathrm{LIG}}\;\pm \;\mathrm{SD}$	$\Delta \Delta G_{\mathrm{RBFE}}^{A\to B}$
C36 (−11.33)	C28 (−9.89)	1.44	−19.51 ± 0.87	−22.45 ± 0.99	2.94
	C38 (−8.33)	3.00	−17.12 ± 2.35	−21.77 ± 1.41	4.58
	C64 (−9.67)	1.66	−19.11 ± 3.26	−10.48 ± 2.95	−8.63
	C73 (−10.40)	0.93	−13.09 ± 0.64	−15.73 ± 2.06	2.64
	C76 (−12.76)	−1.43	−54.20 ± 0.31	−53.97 ± 0.77	−0.23
	C80 (−14.03)	−2.70	−34.22 ± 0.13	−28.88 ± 1.16	−5.34
	C83 (−11.86)	−0.53	−48.29 ± 0.63	−47.34 ± 0.58	−0.97
C70 (−9.53)	C45 (−9.50)	0.03	−19.44 ± 1.02	−25.01 ± 0.26	5.57
	C89 (−10.13)	−0.60	−20.41 ± 0.32	−19.62 ± 0.60	−0.79
	C114 (−10.82)	−1.29	−49.62 ± 1.06	−46.76 ± 3.29	−2.86

$\Delta G_{\mathrm{EXP}}$: experimental binding free energy; $\Delta \Delta G_{\mathrm{EXP}}^{A\to B}$: experimental relative binding free energy; $\Delta G_{\mathrm{COM}}$: free energy changes in complex; $\Delta G_{\mathrm{LIG}}$: free energy changes in isolated form; $\Delta \Delta G_{\mathrm{RBFE}}^{A\to B}$: computed relative binding free energy.

## Data Availability

The data are available within the article or its supplementary information.

## Supplementary Materials

The following supporting information can be downloaded at: https://www.mdpi.com/article/10.3390/molecules28031464/s1. Figure S1: Binding poses comparison of the TAE226 compound between MD and the crystal form; Figure S2: Correlation plot between experimental and computed relative binding free energies; Table S1: MM-PB/GBSA binding energy terms; Table S2: Residue-specific MM-PB/GBSA binding energy decomposition in kcal/mol; Table S3: Dataset compounds and their corresponding pIC_50_ values; Table S4: Randomly drawn table for the test set compounds; Table S5: Statistics of CoMSIA model development; Table S6: Actual vs. predicted pIC_50_ values of CoMFA and CoMSIA (SET-D) models; Table S7: λ parameters to gradually change the ligand interaction from state-A to state-B.

## Abbreviations

SBDD	Structure-based Drug Discovery
FAK	Focal Adhesion Kinase
MD	Molecular Dynamics
3D-QSAR	Three-dimensional Quantitative Structure-Activity Relationship
MM-PB/GBSA	Molecular Mechanics-Poison Boltzmann/Generalized Born Surface Area
FEP	Free Energy Perturbation
CoMFA	Comparative Molecular Field Analysis
CoMSIA	Comparative Molecular Similarity Indices Analysis
ML	Machine Learning
SAR	Structure-Activity Relationship
LJ	Lennard-Jones
BAR	Bennet Acceptance Ratio
ACEPYPE	AnteChamber Python Parser interfacE
AD	Applicability Domain

## References

[1] Liao Y., Liu L., Yang J., Shi Z. (2022). ATX/LPA axis regulates FAK activation, cell proliferation, apoptosis, and motility in human pancreatic cancer cells. In Vitro Cell. Dev. Biol. Anim..

[2] Pomella S., Cassandri M., Braghini M.R., Marampon F., Alisi A., Rota R. (2022). New Insights on the Nuclear Functions and Targeting of FAK in Cancer. Int. J. Mol. Sci..

[3] Le Coq J., Acebrón I., Martin B.R., Navajas P.L., Lietha D. (2022). New insights into FAK structure and function in focal adhesions. J. Cell Sci..

[4] Zhai C., Zhang N., Wang J., Cao M., Luan J., Liu H., Zhang Q., Zhu Y., Xue Y., Li S. (2022). Activation of autophagy induces monocrotaline-induced pulmonary arterial hypertension by FOXM1-mediated FAK phosphorylation. Lung.

[5] Yang J.Y., Woo H.J., Lee P., Kim S.-H. (2022). Induction of Apoptosis and Effect on the FAK/AKT/mTOR Signal Pathway by Evodiamine in Gastric Cancer Cells. Curr. Issues Mol. Biol..

[6] Spallarossa A., Tasso B., Russo E., Villa C., Brullo C. (2022). The Development of FAK Inhibitors: A Five-Year Update. Int. J. Mol. Sci..

[7] Wankowicz S.A., de Oliveira S.H., Hogan D.W., Bedem H.V.D., Fraser J.S. (2022). Ligand binding remodels protein side-chain conformational heterogeneity. Elife.

[8] Singh V.K., Chaurasia H., Mishra R., Srivastava R., Naaz F., Kumar P., Singh R.K. (2022). Docking, ADMET prediction, DFT analysis, synthesis, cytotoxicity, antibacterial screening and QSAR analysis of diarylpyrimidine derivatives. J. Mol. Struct..

[9] Castelli M., Serapian S.A., Marchetti F., Triveri A., Pirota V., Torielli L., Collina S., Doria F., Freccero M., Colombo G. (2021). New perspectives in cancer drug development: Computational advances with an eye to design. RSC Med. Chem..

[10] Wang R., Chen Y., Yang B., Yu S., Zhao X., Zhang C., Hao C., Zhao D., Cheng M. (2020). Design, synthesis, biological evaluation and molecular modeling of novel 1H-pyrrolo[2,3-b]pyridine derivatives as potential anti-tumor agents. Bioorg. Chem..

[11] Wang R., Zhao X., Yu S., Chen Y., Cui H., Wu T., Hao C., Zhao D., Cheng M. (2020). Discovery of 7H-pyrrolo[2,3-d]pyridine derivatives as potent FAK inhibitors: Design, synthesis, biological evaluation and molecular docking study. Bioorg. Chem..

[12] Qu M., Liu Z., Zhao D., Wang C., Zhang J., Tang Z., Liu K., Shu X., Yuan H., Ma X. (2017). Design, synthesis and biological evaluation of sulfonamide-substituted diphenylpyrimidine derivatives (Sul-DPPYs) as potent focal adhesion kinase (FAK) inhibitors with antitumor activity. Bioorg. Med. Chem..

[13] Wang R., Yu S., Zhao X., Chen Y., Yang B., Wu T., Hao C., Zhao D., Cheng M. (2020). Design, synthesis, biological evaluation and molecular docking study of novel thieno[3,2-d]pyrimidine derivatives as potent FAK inhibitors. Eur. J. Med. Chem..

[14] Xie H., Lin X., Zhang Y., Tan F., Chi B., Peng Z., Dong W., An D. (2020). Design, synthesis and biological evaluation of ring-fused pyrazoloamino pyridine/pyrimidine derivatives as potential FAK inhibitors. Bioorg. Med. Chem. Lett..

[15] Halder J., Lin Y.G., Merritt W.M., Spannuth W.A., Nick A.M., Honda T., Kamat A.A., Han L.Y., Kim T.J., Pluquet O. (2007). Therapeutic efficacy of a novel focal adhesion kinase inhibitor TAE226 in ovarian carcinoma. Cancer Res..

[16] Shi Q., Hjelmeland A.B., Keir S.T., Song L., Wickman S., Jackson D., Ohmori O., Bigner D.D., Friedman H.S., Rich J.N. (2007). A novel low-molecular weight inhibitor of focal adhesion kinase, TAE226, inhibits glioma growth. Mol. Carcinog..

[17] Lietha D., Eck M.J. (2008). Crystal structures of the FAK kinase in complex with TAE226 and related bis-anilino pyrimidine inhibitors reveal a helical DFG conformation. PLoS ONE.

[18] Zhou J., Bronowska A., Le Coq J., Lietha D., Gräter F. (2015). Allosteric regulation of focal adhesion kinase by PIP2 and ATP. Biophys. J..

[19] Ghosh S., Cho S.J. (2022). Structural Insights from Molecular Modeling of Isoindolin-1-One Derivatives as PI3Kγ Inhibitors against Gastric Carcinoma. Biomedicines.

[20] Keretsu S., Ghosh S., Cho S.J. (2021). Computer aided designing of novel pyrrolopyridine derivatives as JAK1 inhibitors. Sci. Rep..

[21] Bang S.J., Cho S.J. (2004). Comparative molecular field analysis (CoMFA) and comparative molecular similarity index analysis (CoMSIA) study of mutagen X. Bull. Korean Chem. Soc..

[22] San Juan A.A., Cho S.J. (2007). 3D-QSAR study of microsomal prostaglandin E_2_ synthase (mPGES-1) inhibitors. J. Mol. Model..

[23] Ghosh S., Cho S.J. (2022). Structure–activity relationship and in silico development of c-Met kinase inhibitors. Bull. Korean Chem. Soc..

[24] Ghosh S., Cho S.J. (2022). Binding Studies and Lead Generation of Pteridin-7 (8H)-one Derivatives Targeting FLT3. Int. J. Mol. Sci..

[25] Ghosh S., Keretsu S., Cho S.J. (2021). Computational Modeling of Novel Phosphoinositol-3-kinase γ Inhibitors Using Molecular Docking, Molecular Dynamics, and 3D-QSAR. Bull. Korean Chem. Soc..

[26] Ghosh S., Keretsu S., Cho S.J. (2020). 3D-QSAR, Docking and Molecular Dynamics Simulation Study of C-Glycosylflavones as GSK-3β Inhibitors. J. Chosun Nat. Sci..

[27] Abraham M.J., Murtola T., Schulz R., Páll S., Smith J.C., Hess B., Lindahl E. (2015). GROMACS: High performance molecular simulations through multi-level parallelism from laptops to supercomputers. SoftwareX.

[28] Ghosh S., Keretsu S., Cho S.J. (2021). Molecular Modeling Studies of N-phenylpyrimidine-4-amine Derivatives for Inhibiting FMS-like Tyrosine Kinase-3. Int. J. Mol. Sci..

[29] Ghosh S., Cho S.J. (2022). Comparative binding affinity analysis of dual CDK2/FLT3 inhibitors. Bull. Korean Chem. Soc..

[30] Sousa da Silva A.W., Vranken W.F. (2012). ACPYPE-Antechamber python parser interface. BMC Res. Notes.

[31] Valdés-Tresanco M.S., Valiente P.A., Moreno E. (2021). gmx_MMPBSA: A New Tool to Perform End-State Free Energy Calculations with GROMACS. J. Chem. Theory Comput..

[32] Ghosh S., Keretsu S., Cho S.J. (2021). Designing of the N-ethyl-4-(pyridin-4-yl)benzamide based potent ROCK1 inhibitors using docking, molecular dynamics, and 3D-QSAR. PeerJ.

[33] Todeschini R., Ballabio D., Grisoni F. (2016). Beware of unreliable Q 2! A comparative study of regression metrics for predictivity assessment of QSAR models. J. Chem. Inf. Model..

[34] Veerasamy R., Rajak H., Jain A., Sivadasan S., Varghese C.P., Agrawal R.K. (2011). Validation of QSAR models-strategies and importance. Int. J. Drug Des. Discov..

[35] Abdizadeh R., Hadizadeh F., Abdizadeh T. (2020). QSAR analysis of coumarin-based benzamides as histone deacetylase inhibitors using CoMFA, CoMSIA and HQSAR methods. J. Mol. Struct..

[36] Cournia Z., Allen B., Sherman W. (2017). Relative binding free energy calculations in drug discovery: Recent advances and practical considerations. J. Chem. Inf. Model..

[37] Jung J., Mori T., Kobayashi C., Matsunaga Y., Yoda T., Feig M., Sugita Y. (2015). GENESIS: A hybrid-parallel and multi-scale molecular dynamics simulator with enhanced sampling algorithms for biomolecular and cellular simulations. Wiley Interdiscip. Rev. Comput Mol. Sci..

[38] Huang J., Rauscher S., Nawrocki G., Ran T., Feig M., De Groot B.L., Grubmüller H., MacKerell A.D. (2017). CHARMM36: An improved force field for folded and intrinsically disordered proteins. Biophys. J..

[39] Kim S., Oshima H., Zhang H., Kern N.R., Re S., Lee J., Roux B., Sugita Y., Jiang W., Im W. (2020). CHARMM-GUI free energy calculator for absolute and relative ligand solvation and binding free energy simulations. J. Chem. Theory Comput..

