# Supplementary material for: Three-Dimensional-QSAR and Relative Binding Affinity Estimation of Focal Adhesion Kinase Inhibitors
Source: Molecules. 2023 Feb 2;28(3):1464. doi: 10.3390/molecules28031464 (PMC9919860; doi:10.3390/molecules28031464)
Supplement: Supplementary file 1 [file molecules-28-01464-s001.zip › molecules-2071402-supplementary.pdf]

*Supplementary Information*

# Three-dimensional-QSAR and Relative Binding Affinity Estimation of Focal Adhesion Kinase Inhibitors

**Suparna Ghosh** [1] **and Seung Joo Cho** [1,2,*]

[1] Department of Biomedical Sciences, College of Medicine, Chosun University, Gwangju 501-759, Republic of Korea

[2] Department of Cellular Molecular Medicine, College of Medicine, Chosun University, Gwangju 501-759, Republic of Korea

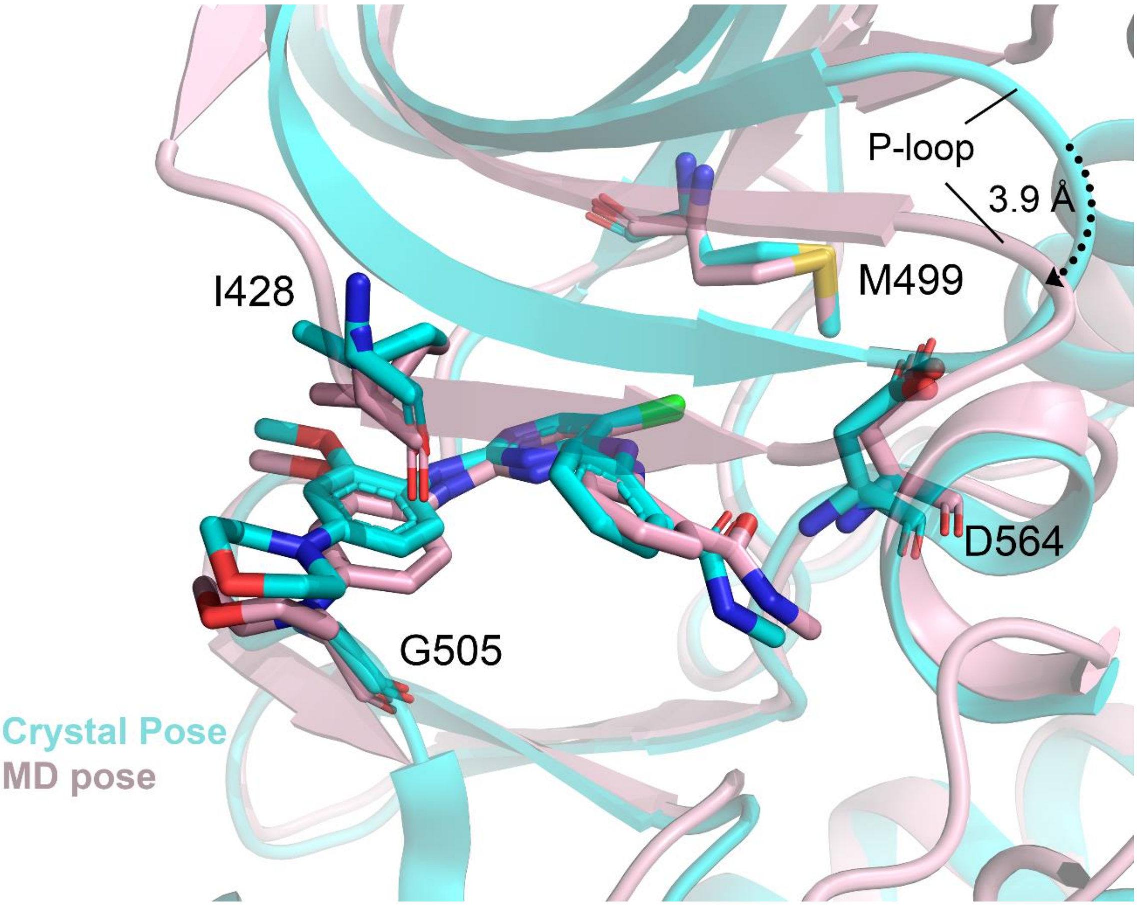


**Figure S1.** Binding poses comparison of TAE226 compound between MD and crystal form. The original crystal complex comprises homodimer of chain A and B. TAE226 is missing from Chain A, although it is present in Chain B. For the MD study, only TAE226 bound was considered.

**Table S1.** MM-PB/GBSA binding energy terms

.

| Binding energy terms | BE in kcal/mol |
|---|---|
| **VDW** | -58.85 ± 2.34 |
| **$E_{EL}$** | -16.96 ± 2.78 |
| **$E_{GB}$** | 29.54 ± 1.89 |
| **$E_{SURF}$** | -6.49 ± 0.16 |
| **$\Delta G_{gas}$** | -75.81 ± 3.70 |
| **$\Delta G_{solv}$** | 23.05 ± 1.82 |

| | |
|---|---|
| **ΔTOTAL** | -52.76 ± 2.95 |
| **TΔS** | 7.51 ± 0.04 |
| **$\Delta G_{bind}$** | -45.25 ± 2.95 |

**Table S2.** Residue-specific MM-PB/GBSA binding energy decomposition in kcal/mol.

| **Residues** | **BE** |
|---|---|
| I428 | -3.25 ± 0.44 |
| V436 | -1.43 ± 0.26 |
| V484 | -0.70 ± 0.15 |
| M499 | -0.69 ± 0.14 |
| L501 | -2.37 ± 0.34 |
| C502 | -1.97 ± 0.53 |
| G505 | -2.25 ± 0.34 |
| L553 | -2.87 ± 0.38 |
| G563 | -1.04 ± 0.42 |
| D564 | -2.61 ± 0.44 |
| L567 | -2.18 ± 0.30 |

**Table S3.** Dataset compounds and their corresponding $pIC_{50}$ values.

| **#Cpd** | **Structure** | **$pIC_{50}$** |
|---|---|---|
| 01 | 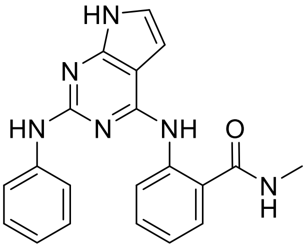 | 6.76 |
| 02 | 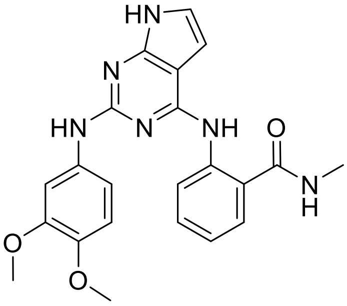 | 7.22 |
| 03 | 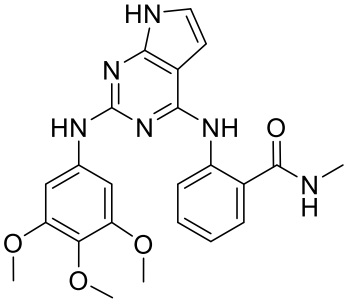 | 7.17 |

| | | |
|---|---|---|
| 04 | 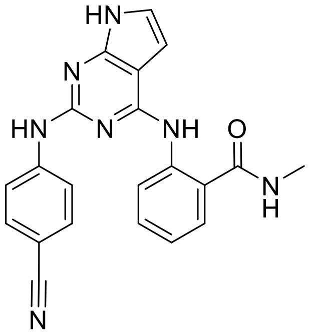 | 7.20 |
| 05 | 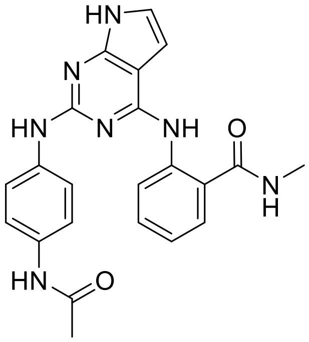 | 7.23 |
| 06 | 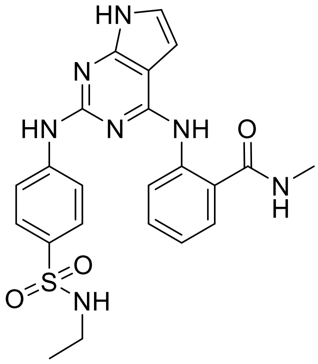 | 7.48 |
| 07 | 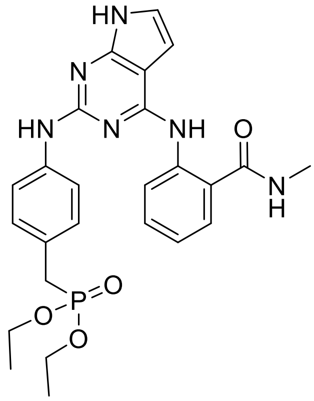 | 6.94 |
| 08 | 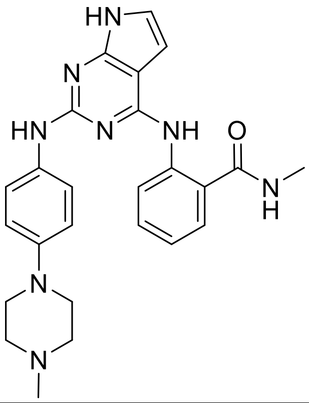 | 7.71 |

| | | |
|---|---|---|
| 09 | 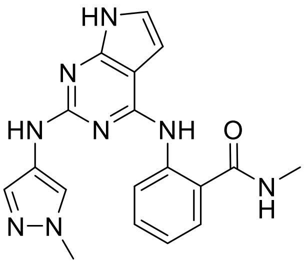 | 7.19 |
| 10 | 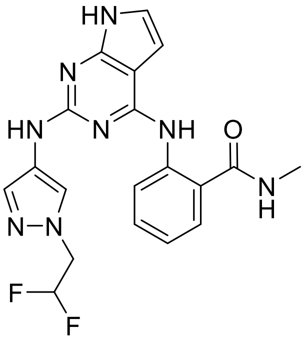 | 7.05 |
| 11 | 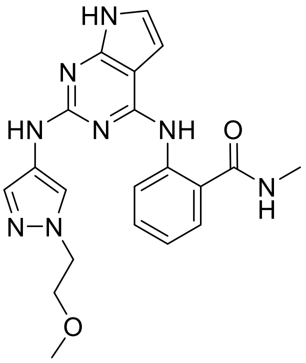 | 7.26 |
| 12 | 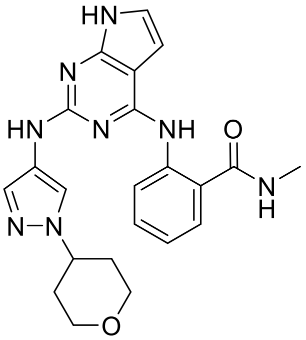 | 7.32 |
| 13 | 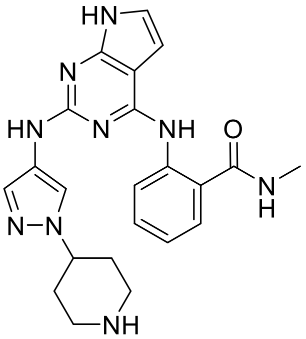 | 7.65 |

| | | |
|---|---|---|
| 14 | 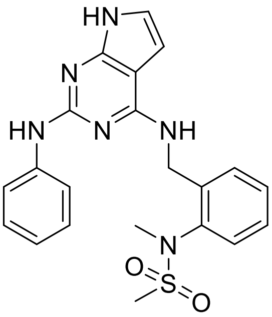 | 7.23 |
| 15 | 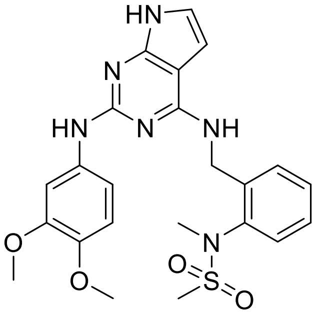 | 7.54 |
| 16 | 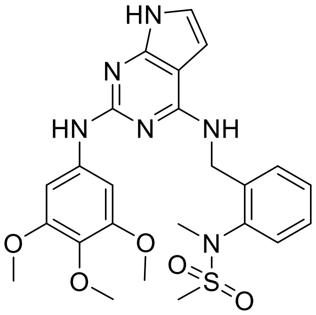 | 7.64 |
| 17 | 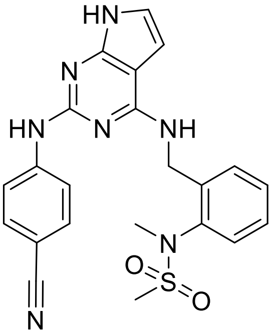 | 7.64 |
| 18 | 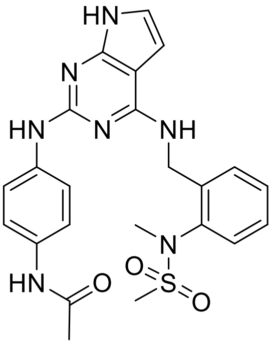 | 7.62 |

| | | |
|---|---|---|
| 19 | 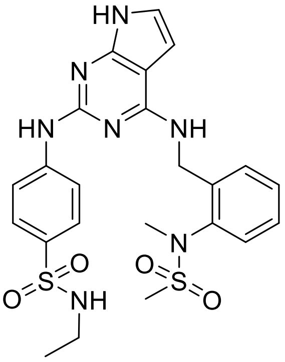 | 7.94 |
| 20 | 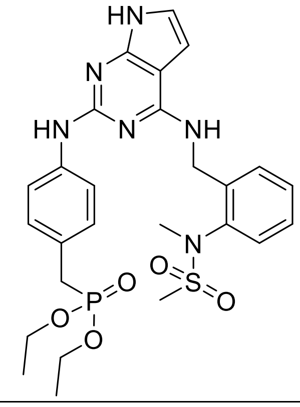 | 7.36 |
| 21 | 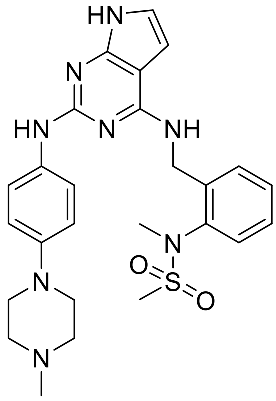 | 7.58 |
| 22 | 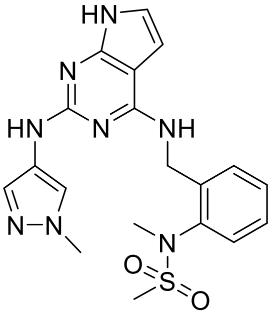 | 7.81 |
| 23 | 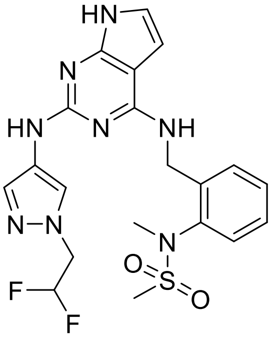 | 7.42 |

| | | |
|---|---|---|
| 24 | 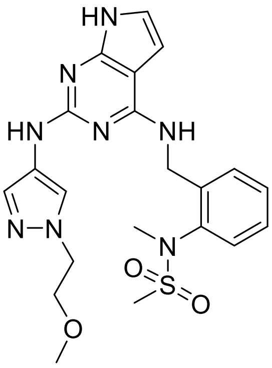 | 8.14 |
| 25 | 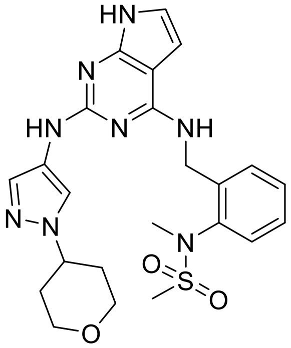 | 7.31 |
| 26 | 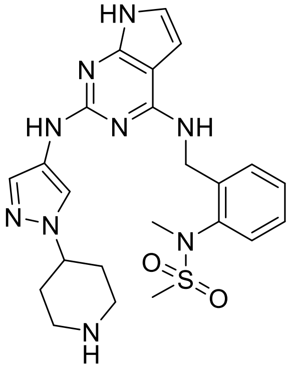 | 7.98 |
| 27 | 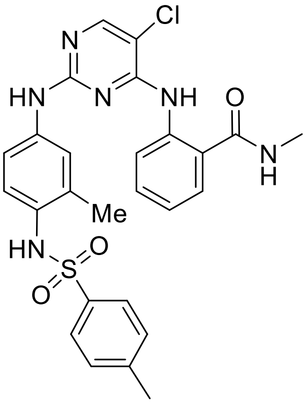 | 7.07 |

| | | |
|---|---|---|
| 28 | 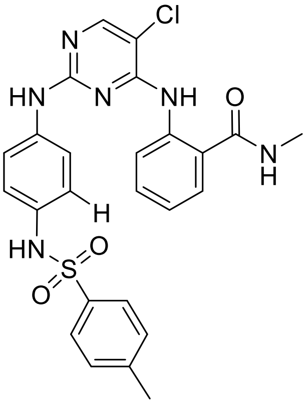 | 7.08 |
| 29 | 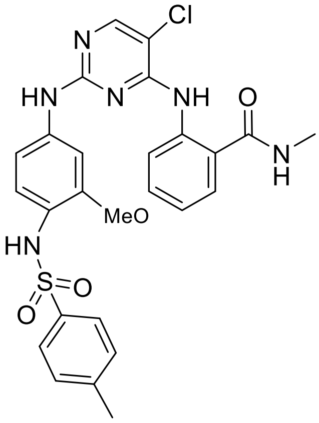 | 6.96 |
| 30 | 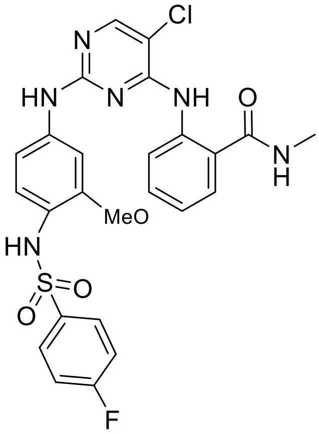 | 7.07 |
| 31 | 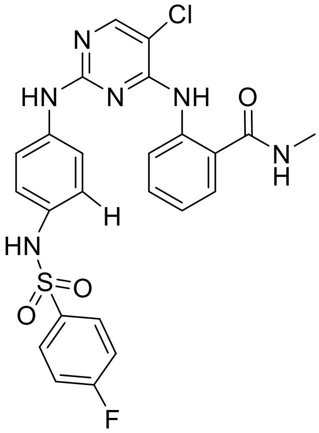 | 7.06 |

| | | |
|---|---|---|
| 32 | 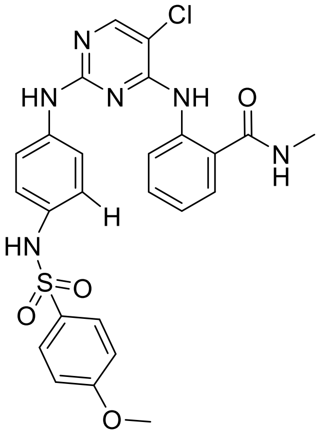 | 6.95 |
| 33 | 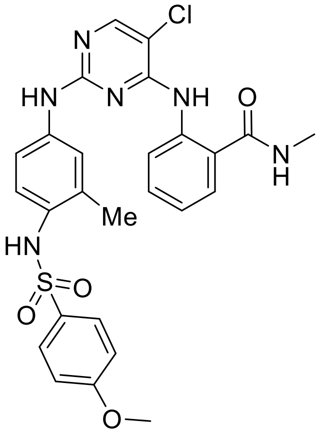 | 6.94 |
| 34 | 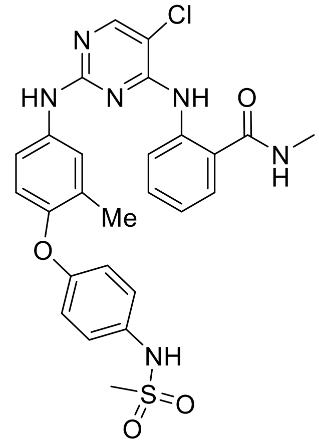 | 6.96 |
| 35 | 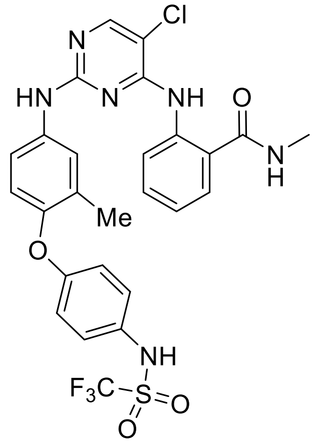 | 6.62 |

| | | |
|---|---|---|
| 36 | 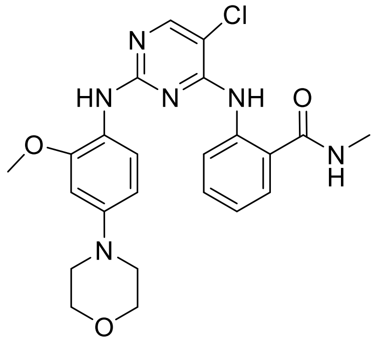 | 8.2 |
| 37 | 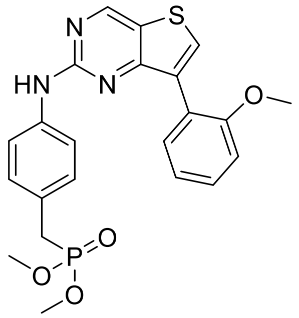 | 6.87 |
| 38 | 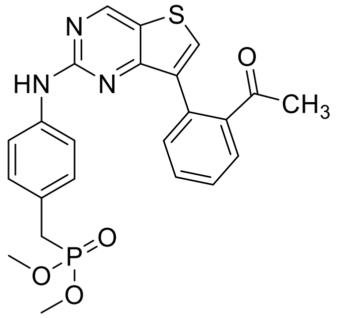 | 5.96 |
| 39 | 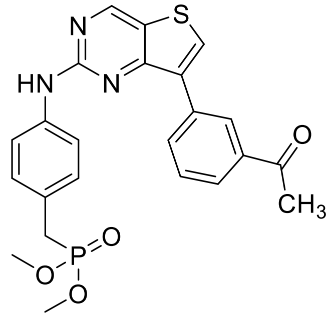 | 6.45 |
| 40 | 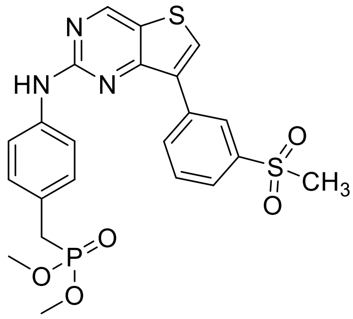 | 6.85 |

| | | |
|---|---|---|
| 41 | 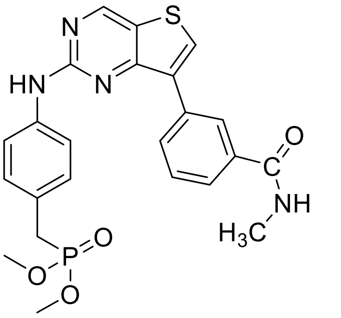 | 6.48 |
| 42 | 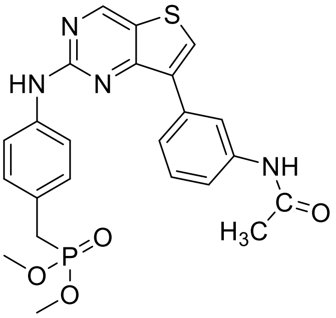 | 6.42 |
| 43 | 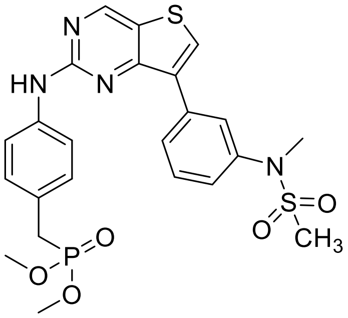 | 6.19 |
| 44 | 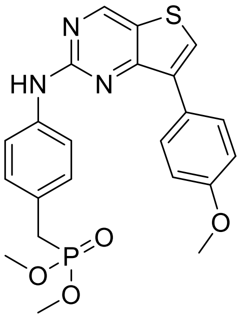 | 5.97 |
| 45 | 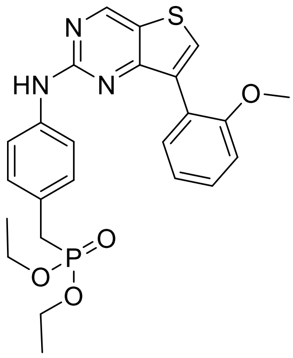 | 6.80 |

| | | |
|---|---|---|
| 46 | 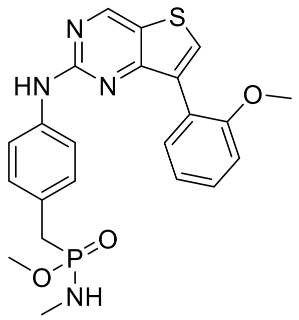 | 6.73 |
| 47 | 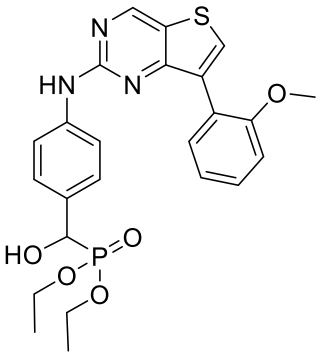 | 6.49 |
| 48 | 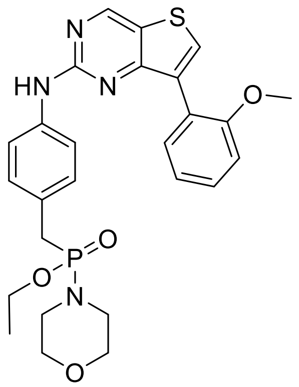 | 6.45 |
| 49 | 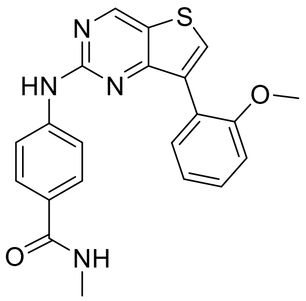 | 7.10 |
| 50 | 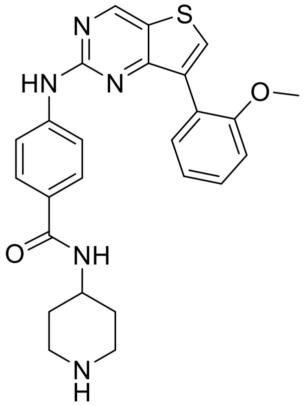 | 7.41 |

| | | |
|---|---|---|
| 51 | 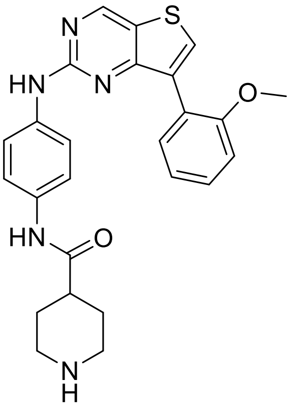 | 7.23 |
| 52 | 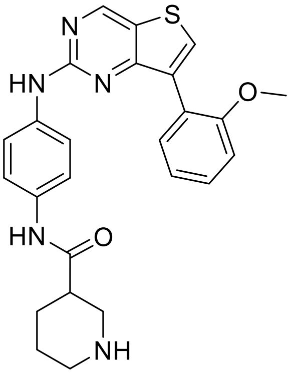 | 6.77 |
| 53 | 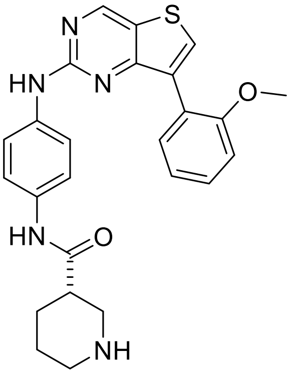 | 6.70 |
| 54 | 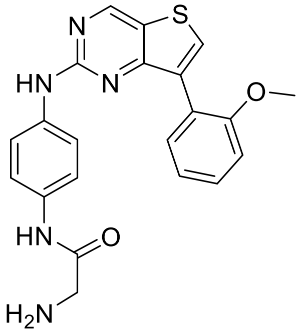 | 6.48 |

| | | |
|---|---|---|
| 55 | 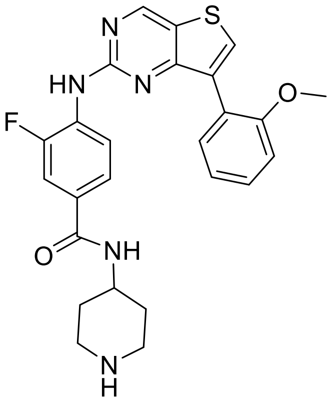 | 7.31 |
| 56 | 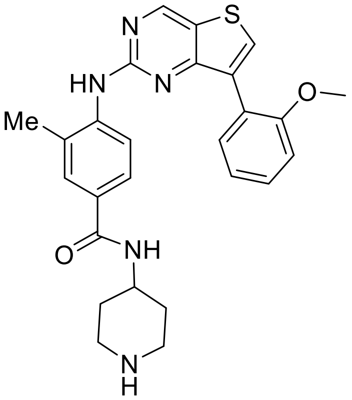 | 7.57 |
| 57 | 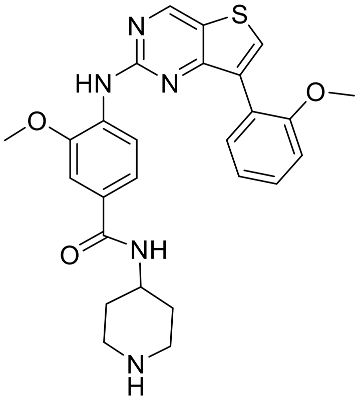 | 7.56 |
| 58 | 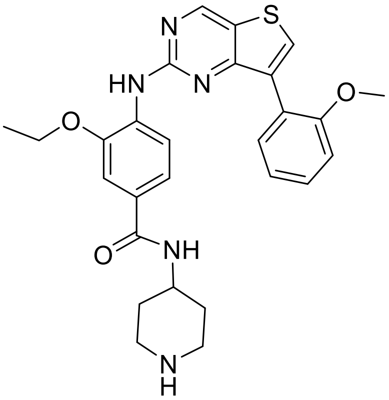 | 6.78 |

| | | |
|---|---|---|
| 59 | 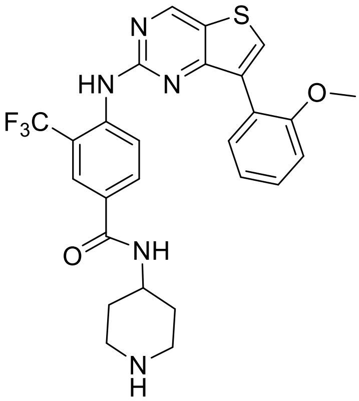 | 6.14 |
| 60 | 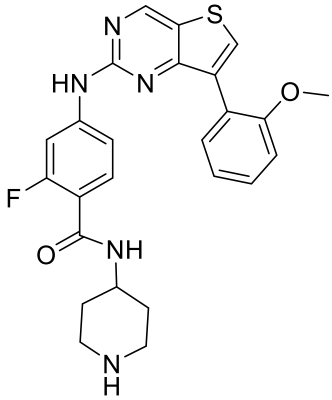 | 7.54 |
| 61 | 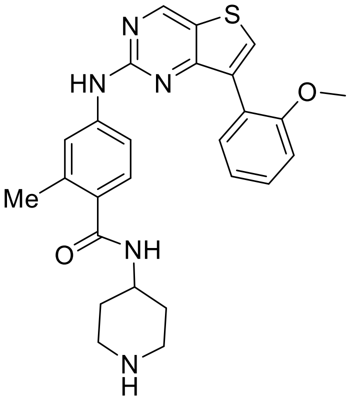 | 7.59 |
| 62 | 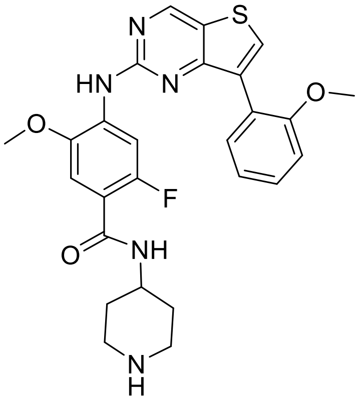 | 7.41 |

| | | |
|---|---|---|
| 63 | 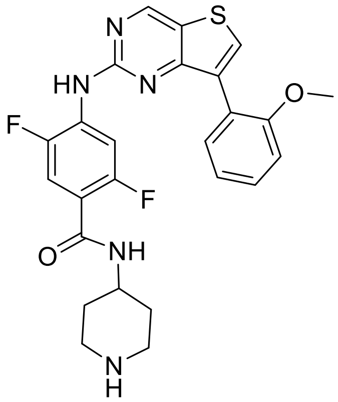 | 7.61 |
| 64 | 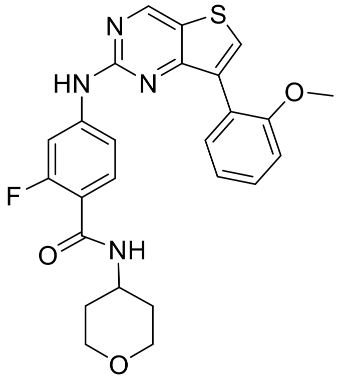 | 6.92 |
| 65 | 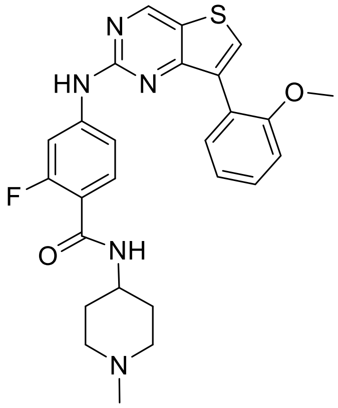 | 7.31 |
| 66 | 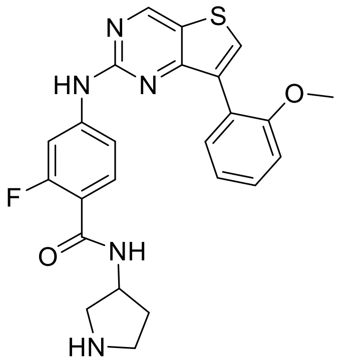 | 6.87 |

| | | |
|---|---|---|
| 67 | 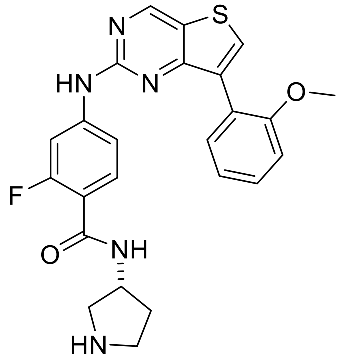 | 7.14 |
| 68 | 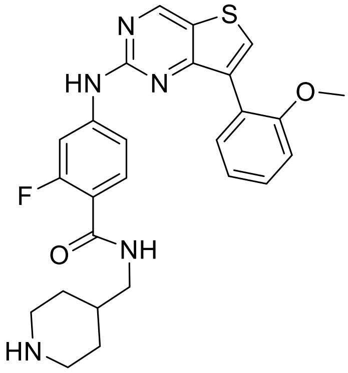 | 6.92 |
| 69 | 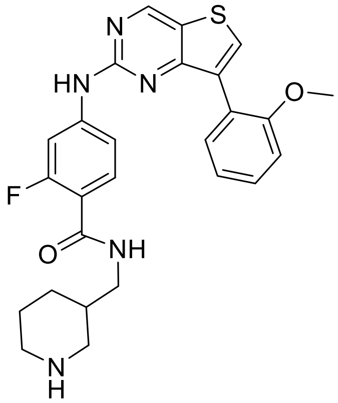 | 6.95 |
| 70 | 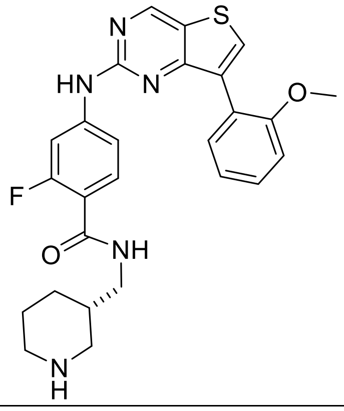 | 6.82 |
| 71 | 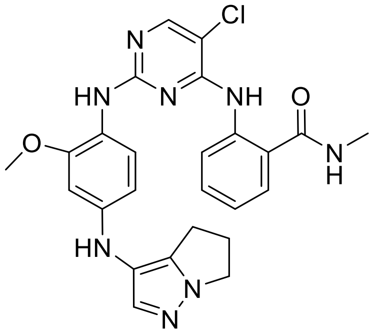 | 9.55 |

| | | |
|---|---|---|
| 72 | 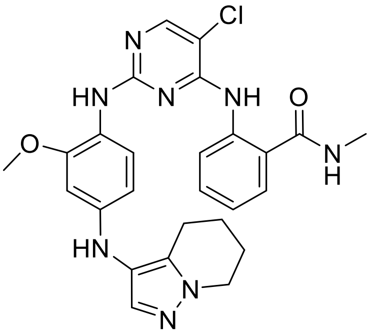 | 9.35 |
| 73 | 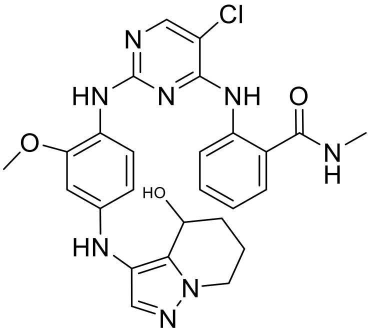 | 7.53 |
| 74 | 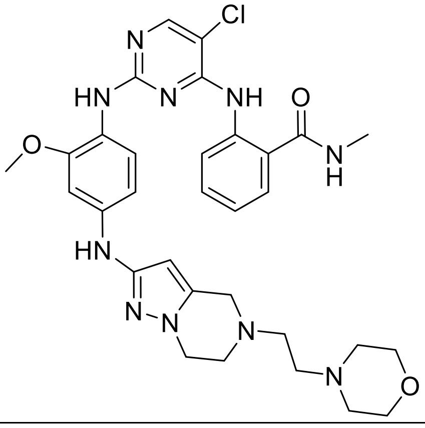 | 8.88 |
| 75 | 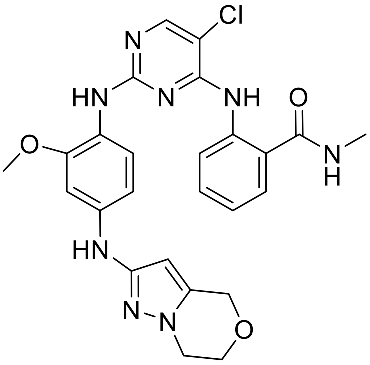 | 8.74 |
| 76 | 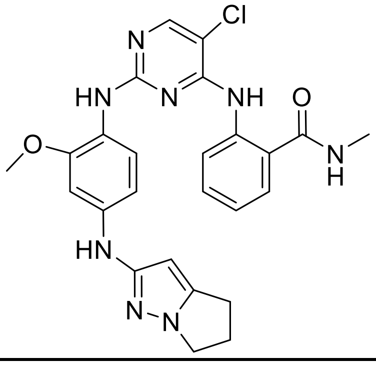 | 9.24 |

| | | |
|---|---|---|
| 77 | 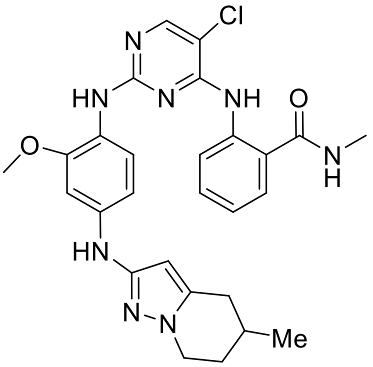 | 9.95 |
| 78 | 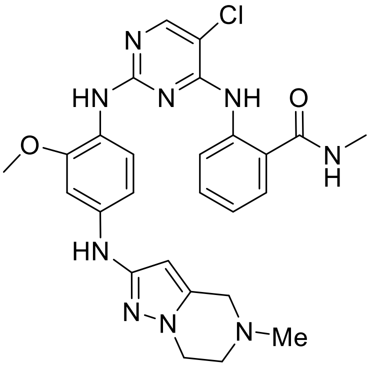 | 8.60 |
| 79 | 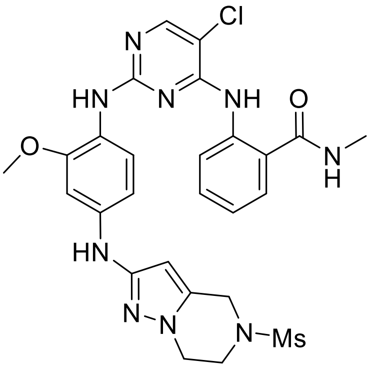 | 9.35 |
| 80 | 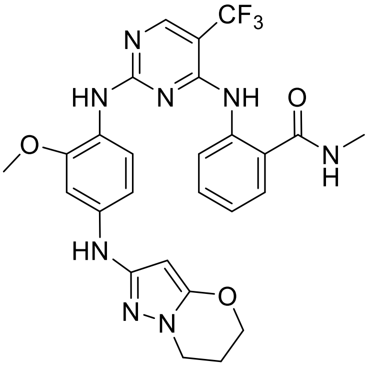 | 9.46 |
| 81 | 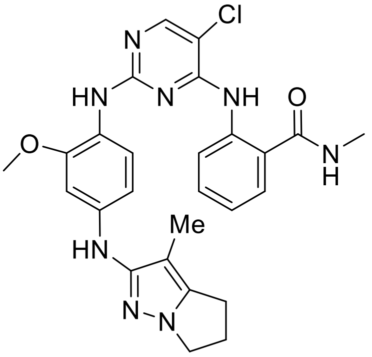 | 10.04 |

| | | |
|---|---|---|
| 82 | 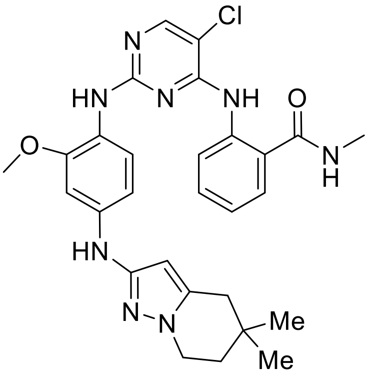 | 8.29 |
| 83 | 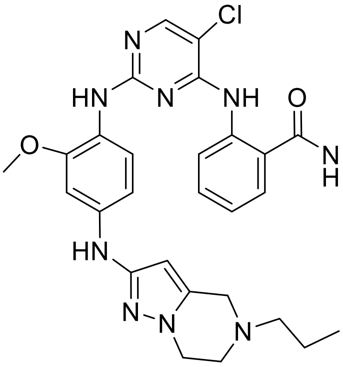 | 8.49 |
| 84 | 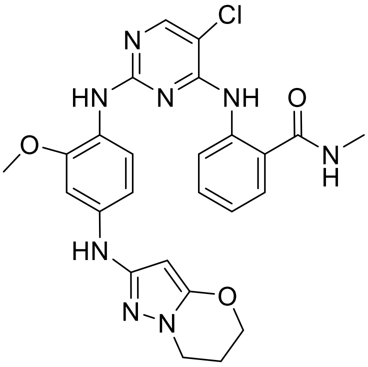 | 9.37 |
| 85 | 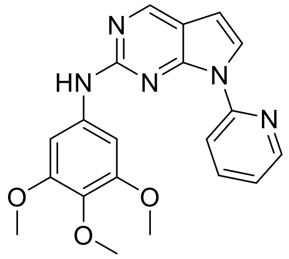 | 6.69 |
| 86 | 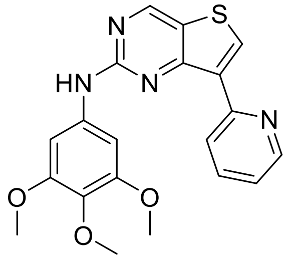 | 7.74 |

| | | |
|---|---|---|
| 87 | 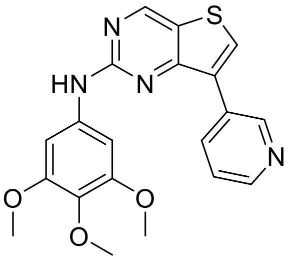 | 7.58 |
| 88 | 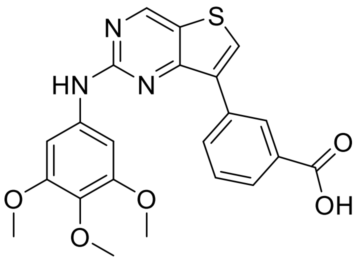 | 7.30 |
| 89 | 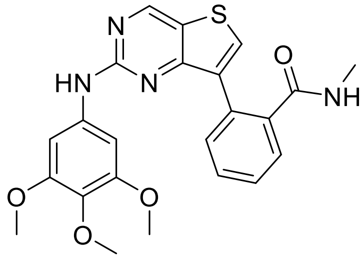 | 7.25 |
| 90 | 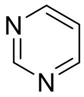 pyrimidine | 6.17 |
| 91 | 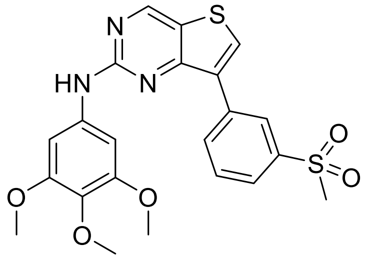 | 7.04 |
| 92 | 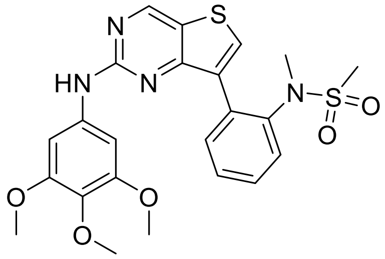 | 7.82 |
| 93 | 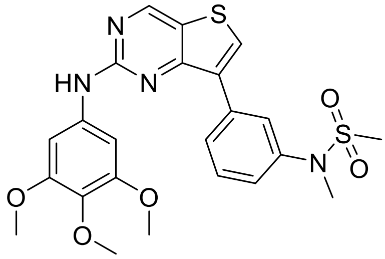 | 7.45 |

| | | |
|---|---|---|
| 94 | 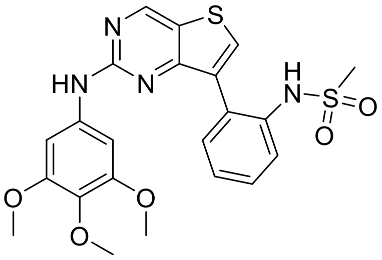 | 7.10 |
| 95 | 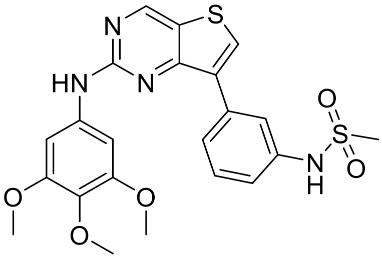 | 8.15 |
| 96 | 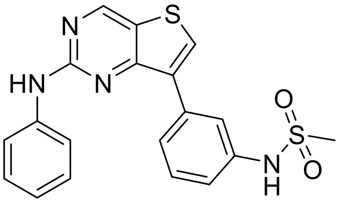 | 7.52 |
| 97 | 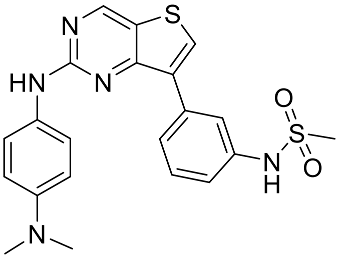 | 7.74 |
| 98 | 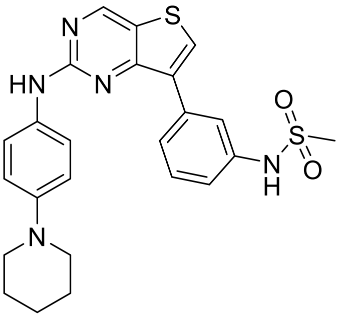 | 7.16 |
| 99 | 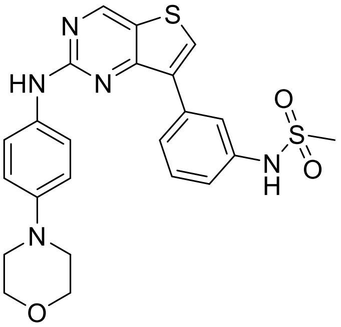 | 8.09 |

| | | |
|---|---|---|
| 100 | 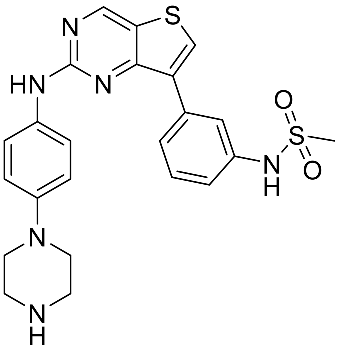 | 7.92 |
| 101 | 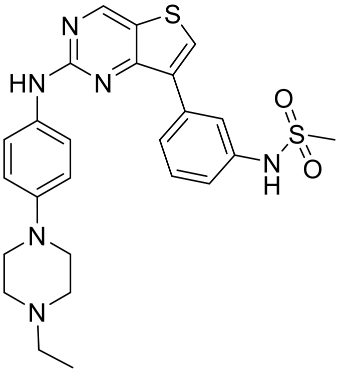 | 8.15 |
| 102 | 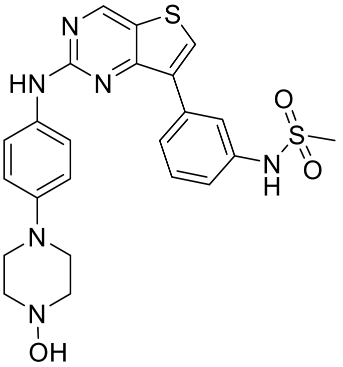 | 7.79 |
| 103 | 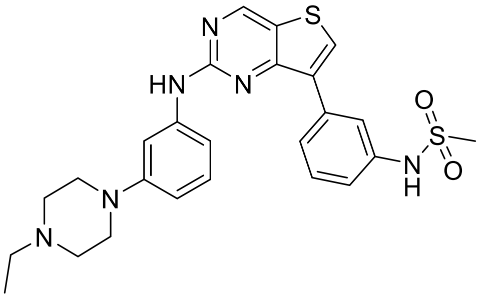 | 7.88 |
| 104 | 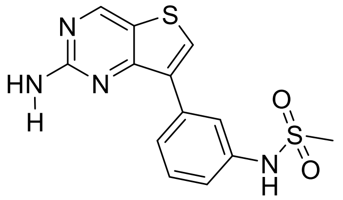 | 5.39 |
| 105 | 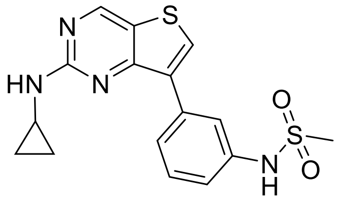 | 5.62 |

| | | |
|---|---|---|
| 106 | 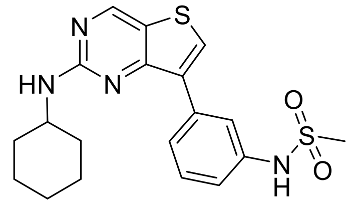 | 6.14 |
| 107 | 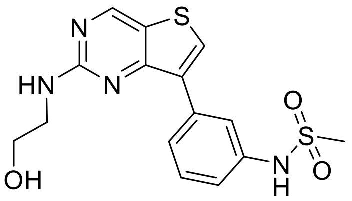 | NA |
| 108 | 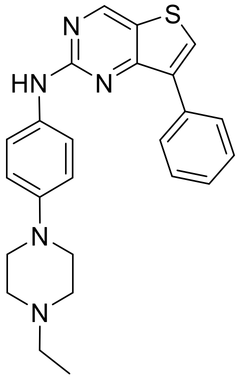 | 8.00 |
| 109 | 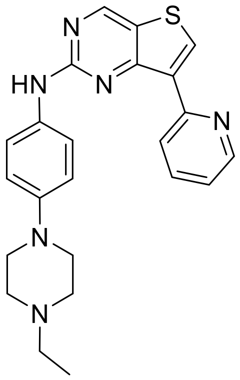 | 7.92 |
| 110 | 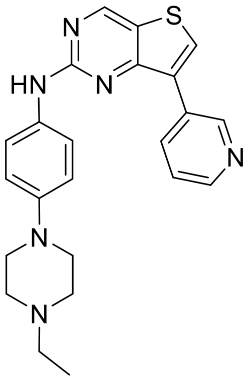 | 7.50 |

| | | |
|---|---|---|
| 111 | 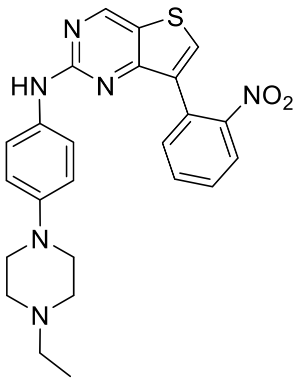 | 6.44 |
| 112 | 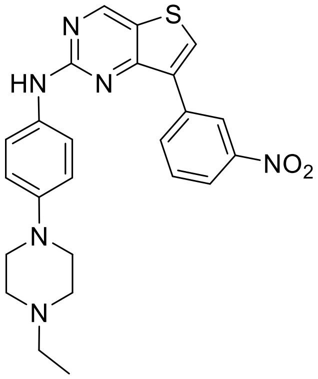 | 7.67 |
| 113 | 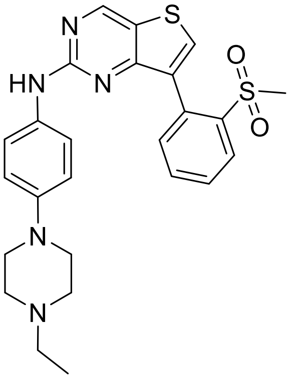 | 6.71 |
| 114 | 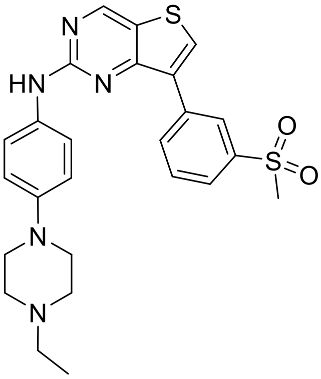 | 7.74 |

| | | |
|---|---|---|
| 115 | 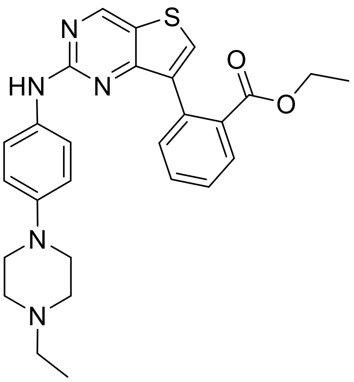 | 5.78 |
| 116 | 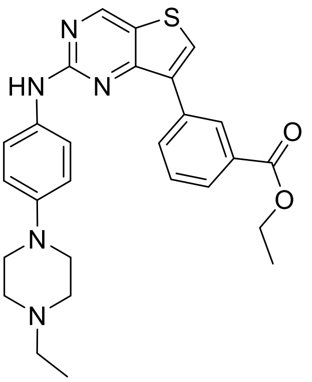 | 6.49 |
| 117 | 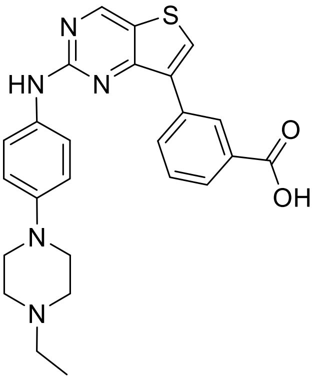 | 7.22 |
| 118 | 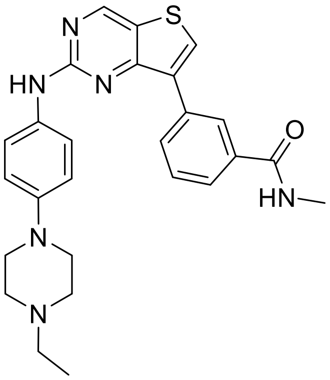 | 7.44 |

| | | |
|---|---|---|
| 119 | 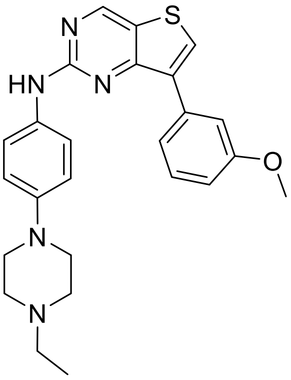 | 7.92 |
| 120 | 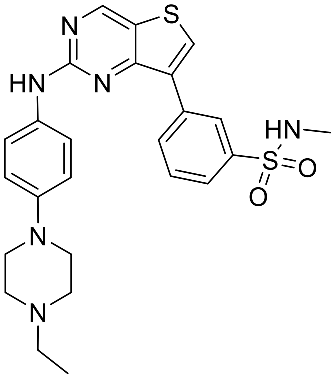 | 7.95 |
| 121 | 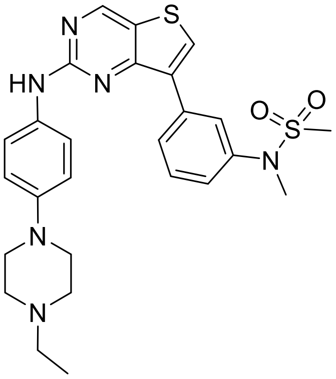 | 7.67 |
| 122 | 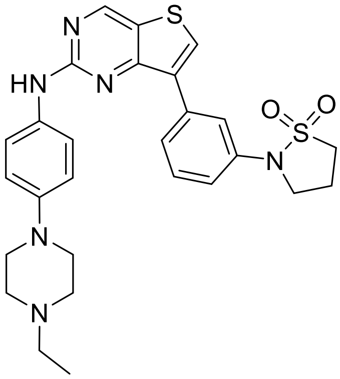 | 7.48 |

| | | |
|---|---|---|
| 123 | 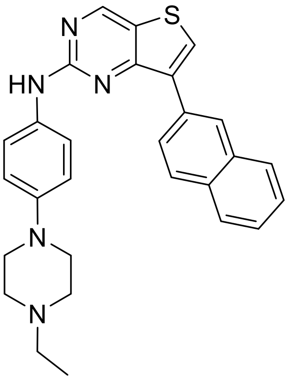 | 6.89 |
| 124 | 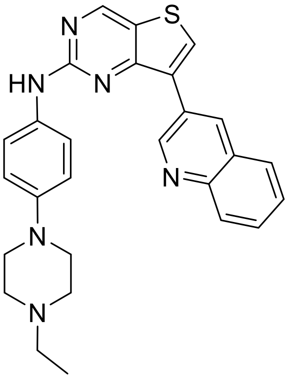 | 7.53 |
| 125 | 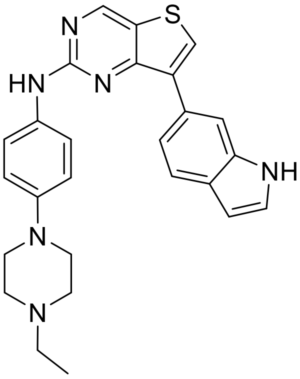 | 7.26 |

**Table S4**. Random draw table for test set compounds.

| High activity | | | Low activity | | | Medium activity | | | |
|---|---|---|---|---|---|---|---|---|---|
| C99 | 8.09 | 0.046511 | C91 | 7.04 | 0.047715 | C94 | 7.1 | 0.067219 | |
| C95 | 8.15 | 0.056668 | C10 | 7.05 | 0.092258 | C56 | 7.57 | 0.070681 | |
| C22 | 7.81 | 0.119098 | C66 | 6.87 | 0.115795 | C50 | 7.41 | 0.094359 | |
| C74 | 8.88 | 0.135938 | C01 | 6.76 | 0.124531 | C110 | 7.5 | 0.11626 | |
| C112 | 7.67 | 0.142158 | C64 | 6.92 | 0.156929 | C93 | 7.45 | 0.146571 | SET-A |
| C97 | 7.74 | 0.154044 | C123 | 6.89 | 0.205567 | C51 | 7.23 | 0.160988 | |
| C120 | 7.95 | 0.170794 | C59 | 6.14 | 0.222062 | C118 | 7.44 | 0.185591 | |
| C119 | 7.92 | 0.184314 | C45 | 6.8 | 0.224221 | C122 | 7.48 | 0.200651 | |
| C82 | 8.29 | 0.188492 | C48 | 6.45 | 0.238903 | C04 | 7.2 | 0.217415 | |
| C36 | 8.2 | 0.188558 | C07 | 6.94 | 0.246183 | C15 | 7.54 | 0.245775 | |
| C76 | 9.24 | 0.19125 | C40 | 6.85 | 0.247239 | C62 | 7.14 | 0.251774 | |
| C19 | 7.94 | 0.270151 | C46 | 6.73 | 0.396101 | C73 | 7.53 | 0.263063 | |
| C26 | 7.98 | 0.27297 | C54 | 6.48 | 0.418428 | C03 | 7.17 | 0.288624 | |
| C83 | 8.49 | 0.285854 | C116 | 6.49 | 0.425414 | C49 | 7.1 | 0.318349 | |
| C121 | 7.67 | 0.291042 | C39 | 6.45 | 0.427529 | C27 | 7.07 | 0.329871 | |
| C16 | 7.64 | 0.32477 | C105 | 5.62 | 0.433339 | C11 | 7.26 | 0.334799 | SET-B |
| C75 | 8.74 | 0.366041 | C44 | 5.97 | 0.449833 | C124 | 7.53 | 0.338937 | |
| C108 | 8 | 0.373527 | C58 | 6.78 | 0.464327 | C12 | 7.32 | 0.3417 | |
| C63 | 7.61 | 0.374259 | C70 | 6.82 | 0.490395 | C14 | 7.23 | 0.368718 | |
| C114 | 7.74 | 0.416158 | C85 | 6.69 | 0.493984 | C89 | 7.25 | 0.375657 | |
| C86 | 7.74 | 0.429647 | C42 | 6.42 | 0.498409 | C98 | 7.16 | 0.476172 | |
| C101 | 8.15 | 0.495314 | C69 | 6.95 | 0.547368 | C65 | 7.31 | 0.484069 | |
| C18 | 7.62 | 0.519801 | C47 | 6.49 | 0.606051 | C25 | 7.31 | 0.484779 | |
| C71 | 9.55 | 0.53617 | C38 | 5.96 | 0.642058 | C57 | 7.56 | 0.557927 | |
| C72 | 9.35 | 0.559578 | C52 | 6.77 | 0.659376 | C28 | 7.08 | 0.574669 | |
| C17 | 7.64 | 0.625503 | C35 | 6.62 | 0.669621 | C20 | 7.36 | 0.596315 | SET-C |
| C80 | 9.46 | 0.633174 | C68 | 6.92 | 0.694384 | C125 | 7.26 | 0.598507 | |
| C24 | 8.14 | 0.750463 | C111 | 6.44 | 0.700532 | C87 | 7.58 | 0.63906 | |
| C84 | 9.37 | 0.75299 | C53 | 6.7 | 0.702626 | C60 | 7.54 | 0.681378 | |
| C13 | 7.65 | 0.805919 | C90 | 6.17 | 0.753446 | C06 | 7.48 | 0.694101 | |
| C109 | 7.92 | 0.817795 | C115 | 5.78 | 0.758656 | C96 | 7.52 | 0.722374 | |
| C102 | 7.79 | 0.849353 | C32 | 6.95 | 0.798334 | C21 | 7.58 | 0.770724 | |
| C61 | 7.59 | 0.852992 | C104 | 5.39 | 0.800923 | C117 | 7.22 | 0.820879 | |
| C78 | 8.6 | 0.909932 | C33 | 6.94 | 0.810285 | C23 | 7.42 | 0.844675 | |
| C81 | 10.04 | 0.922773 | C34 | 6.96 | 0.823336 | C31 | 7.06 | 0.849694 | |
| C92 | 7.82 | 0.926264 | C37 | 6.87 | 0.827899 | C05 | 7.23 | 0.855154 | SET-D |
| C100 | 7.92 | 0.930799 | C113 | 6.71 | 0.847475 | C02 | 7.22 | 0.855431 | |
| C103 | 7.88 | 0.958278 | C29 | 6.96 | 0.848348 | C67 | 7.14 | 0.870785 | |
| C08 | 7.71 | 0.980281 | C43 | 6.19 | 0.866393 | C30 | 7.07 | 0.922571 | |
| C79 | 9.35 | 0.981901 | C106 | 6.14 | 0.985245 | C55 | 7.31 | 0.937029 | |
| C77 | 9.95 | 0.987713 | C41 | 6.48 | 0.994829 | C88 | 7.3 | 0.940625 | |
| | | | | | | C09 | 7.19 | 0.94266 | |

**Table S5.** Statistics of CoMSIA model development.

| CoMSIA | $q^2$ | ONC | SEP | $r^2$ | SEE | F-value | Field Contribution | | | | |
|---|---|---|---|---|---|---|---|---|---|---|---|
| | | | | | | | S | E | H | A | D |
| **S** | **0.476** | **2** | **0.615** | **0.608** | **0.532** | 69.815 | 100 | - | - | - | - |
| E | 0.543 | 3 | 0.577 | 0.672 | 0.489 | 60.786 | - | 100 | - | - | - |
| H | 0.462 | 4 | 0.630 | 0.710 | 0.463 | 53.777 | - | - | 100 | - | - |
| A | 0.412 | 2 | 0.651 | 0.527 | 0.584 | 50.123 | - | - | - | 100 | - |
| D | 0.475 | 5 | 0.626 | 0.711 | 0.465 | 42.738 | - | - | - | - | 100 |
| SE | 0.552 | 6 | 0.582 | 0.853 | 0.333 | 82.997 | 31.2 | 68.8 | - | - | - |
| EH | 0.533 | 6 | 0.594 | 0.854 | 0.332 | 83.767 | - | 62.7 | 37.3 | - | - |
| EA | 0.531 | 2 | 0.582 | 0.656 | 0.498 | 85.688 | - | 59.1 | - | 40.9 | - |
| ED | 0.626 | 6 | 0.531 | 0.838 | 0.352 | 73.143 | - | 59.4 | - | - | 40.6 |
| SH | 0.488 | 2 | 0.608 | 0.641 | 0.509 | 80.493 | 49.9 | - | 50.1 | - | - |
| SA | 0.476 | 5 | 0.626 | 0.786 | 0.400 | 63.954 | 41.1 | - | - | 59.9 | - |
| SD | 0.544 | 4 | 0.580 | 0.749 | 0.430 | 65.747 | 42.8 | - | - | - | 57.2 |
| HA | 0.426 | 5 | 0.654 | 0.760 | 0.423 | 55.022 | - | - | 46.9 | 53.1 | - |
| HD | 0.575 | 4 | 0.560 | 0.768 | 0.414 | 72.690 | - | - | 48.1 | - | 51.9 |
| AD | 0.487 | 3 | 0.612 | 0.666 | 0.494 | 59.212 | - | - | - | 54.6 | 45.4 |
| SHE | 0.556 | 6 | 0.579 | 0.877 | 0.305 | 102.032 | 21.2 | 49.9 | 29.3 | - | - |
| SEA | 0.561 | 6 | 0.576 | 0.864 | 0.320 | 91.420 | 20.4 | 47.0 | - | 32.6 | - |
| SED | 0.656 | 6 | 0.510 | 0.862 | 0.323 | 89.719 | 18.7 | 46.1 | - | - | 35.2 |
| EHA | 0.543 | 6 | 0.588 | 0.853 | 0.333 | 83.432 | - | 44.6 | 25.0 | 30.3 | - |

| | | | | | | | | | | | |
|---|---|---|---|---|---|---|---|---|---|---|---|
| EHD | 0.631 | 6 | 0.528 | 0.863 | 0.322 | 90.298 | - | 42.7 | 23.2 | - | 34.1 |
| SHA | 0.490 | 5 | 0.617 | 0.811 | 0.376 | 74.442 | 25.6 | - | 33.9 | 40.6 | - |
| SHD | 0.574 | 4 | 0.561 | 0.783 | 0.400 | 79.243 | 23.9 | - | 34.9 | - | 41.2 |
| EAD | 0.602 | 5 | 0.545 | 0.817 | 0.369 | 77.741 | - | 43.6 | - | 26.5 | 29.9 |
| HAD | 0.540 | 5 | 0.586 | 0.801 | 0.385 | 70.045 | - | - | 32.9 | 32.2 | 34.9 |
| SEHD | 0.639 | 6 | 0.522 | 0.874 | 0.309 | 99.200 | 14.2 | 35.8 | 19.3 | - | 30.6 |
| SEHA | 0.559 | 6 | 0.577 | 0.873 | 0.310 | 98.318 | 15.5 | 37.0 | 20.5 | 27.0 | - |
| SEAD | 0.639 | 6 | 0.522 | 0.863 | 0.321 | 90.442 | 14.9 | 34.9 | - | 23.9 | 26.3 |
| EHAD | 0.620 | 6 | 0.536 | 0.863 | 0.322 | 90.308 | - | 33.8 | 18.9 | 21.6 | 25.8 |
| SHAD | 0.584 | 5 | 0.557 | 0.827 | 0.359 | 83.193 | 18.0 | - | 25.3 | 27.2 | 29.6 |
| SEHAD | 0.639 | 6 | 0.522 | 0.875 | 0.307 | 100.545 | 12.0 | 28.7 | 15.7 | 19.9 | 23.7 |

(S: Steric; E: Electrostatic; H: Hydrophobic; A: H-bond acceptor; D: H-bond donor).

**Table S6.** Actual vs. predicted $pIC_{50}$ values of CoMFA and CoMSIA (SET-D) models.

| #Cpd | CoMFA | | | CoMSIA (SED) | | |
|---|---|---|---|---|---|---|
| | Actual pIC50 | Predicted pIC50 | Residuals | Actual pIC50 | Predicted pIC50 | Residuals |
| C105 | 5.62 | 5.777 | -0.157 | 5.62 | 5.962 | -0.342 |
| C115 | 5.78 | 6.415 | -0.635 | 5.78 | 6.592 | -0.812 |
| C38 | 5.96 | 6.966 | -1.006 | 5.96 | 6.105 | -0.145 |
| C44 | 5.97 | 7.492 | -1.522 | 5.97 | 6.622 | -0.652 |
| C59 | 6.14 | 7.428 | -1.288 | 6.14 | 7.506 | -1.366 |
| C90 | 6.17 | 6.305 | -0.135 | 6.17 | 6.658 | -0.488 |
| C42 | 6.42 | 6.604 | -0.184 | 6.42 | 6.466 | -0.046 |
| C111 | 6.44 | 6.381 | 0.059 | 6.44 | 6.702 | -0.262 |
| C39 | 6.45 | 6.251 | 0.199 | 6.45 | 6.402 | 0.048 |
| C48 | 6.45 | 6.5 | -0.05 | 6.45 | 6.165 | 0.285 |
| C54 | 6.48 | 6.29 | 0.19 | 6.48 | 6.832 | -0.352 |
| C47 | 6.49 | 6.683 | -0.193 | 6.49 | 6.237 | 0.253 |
| C116 | 6.49 | 6.626 | -0.136 | 6.49 | 7.074 | -0.584 |
| C35 | 6.62 | 6.863 | -0.243 | 6.62 | 6.789 | -0.169 |
| C85 | 6.69 | 7.065 | -0.375 | 6.69 | 7.414 | -0.724 |
| C53 | 6.7 | 6.555 | 0.145 | 6.7 | 6.84 | -0.14 |
| C46 | 6.73 | 6.657 | 0.073 | 6.73 | 6.727 | 0.003 |
| C01 | 6.76 | 7.08 | -0.32 | 6.76 | 6.815 | -0.055 |
| C52 | 6.77 | 6.887 | -0.117 | 6.77 | 6.878 | -0.108 |
| C58 | 6.78 | 7.869 | -1.089 | 6.78 | 8.052 | -1.272 |
| C45 | 6.8 | 6.796 | 0.004 | 6.8 | 6.363 | 0.437 |
| C70 | 6.82 | 7.204 | -0.384 | 6.82 | 7.105 | -0.285 |
| C40 | 6.85 | 6.623 | 0.227 | 6.85 | 6.442 | 0.408 |
| C66 | 6.87 | 7 | -0.13 | 6.87 | 7.131 | -0.261 |
| C123 | 6.89 | 7.283 | -0.393 | 6.89 | 7.339 | -0.449 |
| C64 | 6.92 | 7.152 | -0.232 | 6.92 | 7.001 | -0.081 |
| C68 | 6.92 | 6.898 | 0.022 | 6.92 | 6.846 | 0.074 |
| C07 | 6.94 | 7.044 | -0.104 | 6.94 | 7.192 | -0.252 |
| C69 | 6.95 | 6.904 | 0.046 | 6.95 | 6.706 | 0.244 |
| C91 | 7.04 | 6.999 | 0.041 | 7.04 | 6.954 | 0.086 |
| C10 | 7.05 | 7.255 | -0.205 | 7.05 | 7.154 | -0.104 |
| C27 | 7.07 | 7.168 | -0.098 | 7.07 | 7.123 | -0.053 |
| C28 | 7.08 | 7 | 0.08 | 7.08 | 6.984 | 0.096 |
| C94 | 7.1 | 6.95 | 0.15 | 7.1 | 7.024 | 0.076 |
| C49 | 7.1 | 7.022 | 0.078 | 7.1 | 6.841 | 0.259 |
| C62 | 7.14 | 7.77 | -0.63 | 7.14 | 7.611 | -0.471 |
| C98 | 7.16 | 7.924 | -0.764 | 7.16 | 7.33 | -0.17 |
| C03 | 7.17 | 7.562 | -0.392 | 7.17 | 7.407 | -0.237 |
| C09 | 7.19 | 7.374 | -0.184 | 7.19 | 7.229 | -0.039 |
| C04 | 7.2 | 7.146 | 0.054 | 7.2 | 7.089 | 0.111 |
| C51 | 7.23 | 6.982 | 0.248 | 7.23 | 7.11 | 0.12 |
| C14 | 7.23 | 7.206 | 0.024 | 7.23 | 7.128 | 0.102 |
| C89 | 7.25 | 6.607 | 0.643 | 7.25 | 6.59 | 0.66 |
| C125 | 7.26 | 7.408 | -0.148 | 7.26 | 7.382 | -0.122 |
| C11 | 7.26 | 7.403 | -0.143 | 7.26 | 6.856 | 0.404 |

| | | | | | | |
|---|---|---|---|---|---|---|
| C25 | 7.31 | 7.478 | -0.168 | 7.31 | 7.529 | -0.219 |
| C65 | 7.31 | 7.24 | 0.07 | 7.31 | 7.064 | 0.246 |
| C12 | 7.32 | 7.163 | 0.157 | 7.32 | 7.498 | -0.178 |
| C20 | 7.36 | 7.365 | -0.005 | 7.36 | 7.333 | 0.027 |
| C50 | 7.41 | 7.182 | 0.228 | 7.41 | 7.406 | 0.004 |
| C118 | 7.44 | 7.75 | -0.31 | 7.44 | 7.338 | 0.102 |
| C93 | 7.45 | 8.25 | -0.8 | 7.45 | 7.67 | -0.22 |
| C06 | 7.48 | 7.416 | 0.064 | 7.48 | 7.745 | -0.265 |
| C122 | 7.48 | 6.718 | 0.762 | 7.48 | 7.295 | 0.185 |
| C110 | 7.5 | 7.654 | -0.154 | 7.5 | 7.532 | -0.032 |
| C96 | 7.52 | 8.198 | -0.678 | 7.52 | 7.227 | 0.293 |
| C73 | 7.53 | 9.3 | -1.77 | 7.53 | 9.108 | -1.578 |
| C124 | 7.53 | 7.418 | 0.112 | 7.53 | 7.177 | 0.353 |
| C60 | 7.54 | 7.236 | 0.304 | 7.54 | 7.453 | 0.087 |
| C15 | 7.54 | 7.5 | 0.04 | 7.54 | 7.469 | 0.071 |
| C57 | 7.56 | 7.976 | -0.416 | 7.56 | 8.009 | -0.449 |
| C56 | 7.57 | 7.527 | 0.043 | 7.57 | 7.759 | -0.189 |
| C87 | 7.58 | 7.637 | -0.057 | 7.58 | 7.529 | 0.051 |
| C63 | 7.61 | 7.079 | 0.531 | 7.61 | 7.275 | 0.335 |
| C18 | 7.62 | 7.411 | 0.209 | 7.62 | 7.647 | -0.027 |
| C17 | 7.64 | 7.21 | 0.43 | 7.64 | 7.174 | 0.466 |
| C16 | 7.64 | 7.542 | 0.098 | 7.64 | 7.518 | 0.122 |
| C13 | 7.65 | 7.328 | 0.322 | 7.65 | 7.461 | 0.189 |
| C112 | 7.67 | 7.415 | 0.255 | 7.67 | 7.065 | 0.605 |
| C121 | 7.67 | 7.618 | 0.052 | 7.67 | 7.683 | -0.013 |
| C114 | 7.74 | 7.707 | 0.033 | 7.74 | 7.38 | 0.36 |
| C86 | 7.74 | 7.286 | 0.454 | 7.74 | 7.484 | 0.256 |
| C97 | 7.74 | 7.55 | 0.19 | 7.74 | 7.839 | -0.099 |
| C22 | 7.81 | 7.383 | 0.427 | 7.81 | 7.232 | 0.578 |
| C119 | 7.92 | 7.839 | 0.081 | 7.92 | 7.553 | 0.367 |
| C109 | 7.92 | 7.279 | 0.641 | 7.92 | 7.488 | 0.432 |
| C19 | 7.94 | 7.496 | 0.444 | 7.94 | 7.266 | 0.674 |
| C120 | 7.95 | 7.512 | 0.438 | 7.95 | 7.897 | 0.053 |
| C26 | 7.98 | 7.616 | 0.364 | 7.98 | 7.918 | 0.062 |
| C108 | 8 | 7.576 | 0.424 | 8 | 7.355 | 0.645 |
| C99 | 8.09 | 7.995 | 0.095 | 8.09 | 7.817 | 0.273 |
| C24 | 8.14 | 7.249 | 0.891 | 8.14 | 7.425 | 0.715 |
| C101 | 8.15 | 8.236 | -0.086 | 8.15 | 7.869 | 0.281 |
| C95 | 8.15 | 8.35 | -0.2 | 8.15 | 8.458 | -0.308 |
| C36 | 8.2 | 8.332 | -0.132 | 8.2 | 7.951 | 0.249 |
| C82 | 8.29 | 8.845 | -0.555 | 8.29 | 9.14 | -0.85 |
| C83 | 8.49 | 8.409 | 0.081 | 8.49 | 8.613 | -0.123 |
| C75 | 8.74 | 8.846 | -0.106 | 8.74 | 8.593 | 0.147 |
| C74 | 8.88 | 8.911 | -0.031 | 8.88 | 8.989 | -0.109 |
| C76 | 9.24 | 8.916 | 0.324 | 9.24 | 8.836 | 0.404 |
| C72 | 9.35 | 9.362 | -0.012 | 9.35 | 9.248 | 0.102 |
| C84 | 9.37 | 9.363 | 0.007 | 9.37 | 9.237 | 0.133 |
| C80 | 9.46 | 9.475 | -0.015 | 9.46 | 9.262 | 0.198 |
| C71 | 9.55 | 8.86 | 0.69 | 9.55 | 8.774 | 0.776 |
| C104 | 5.39 | 5.672 | -0.282 | 5.39 | 5.403 | -0.013 |
| C106 | 6.14 | 6.452 | -0.312 | 6.14 | 6.272 | -0.132 |
| C43 | 6.19 | 6.276 | -0.086 | 6.19 | 6.416 | -0.226 |
| C41 | 6.48 | 6.752 | -0.272 | 6.48 | 6.434 | 0.046 |
| C113 | 6.71 | 6.471 | 0.239 | 6.71 | 6.641 | 0.069 |
| C37 | 6.87 | 6.544 | 0.326 | 6.87 | 6.402 | 0.468 |
| C33 | 6.94 | 7.027 | -0.087 | 6.94 | 7.089 | -0.149 |
| C32 | 6.95 | 6.848 | 0.102 | 6.95 | 6.932 | 0.018 |
| C34 | 6.96 | 6.948 | 0.012 | 6.96 | 6.828 | 0.132 |
| C29 | 6.96 | 7.258 | -0.298 | 6.96 | 7.068 | -0.108 |
| C31 | 7.06 | 6.936 | 0.124 | 7.06 | 6.859 | 0.201 |
| C30 | 7.07 | 7.164 | -0.094 | 7.07 | 6.921 | 0.149 |
| C67 | 7.14 | 7.198 | -0.058 | 7.14 | 7.195 | -0.055 |

| | | | | | | |
|---|---|---|---|---|---|---|
| C117 | 7.22 | 7.115 | 0.105 | 7.22 | 7.435 | -0.215 |
| C02 | 7.22 | 7.164 | 0.056 | 7.22 | 7.208 | 0.012 |
| C05 | 7.23 | 7.36 | -0.13 | 7.23 | 7.685 | -0.455 |
| C88 | 7.3 | 7.427 | -0.127 | 7.3 | 7.081 | 0.219 |
| C55 | 7.31 | 7.241 | 0.069 | 7.31 | 7.549 | -0.239 |
| C23 | 7.42 | 7.379 | 0.041 | 7.42 | 7.376 | 0.044 |
| C21 | 7.58 | 7.716 | -0.136 | 7.58 | 8.157 | -0.577 |
| C61 | 7.59 | 7.487 | 0.103 | 7.59 | 7.747 | -0.157 |
| C08 | 7.71 | 7.798 | -0.088 | 7.71 | 8.06 | -0.35 |
| C102 | 7.79 | 8.102 | -0.312 | 7.79 | 7.827 | -0.037 |
| C92 | 7.82 | 6.909 | 0.911 | 7.82 | 7.033 | 0.787 |
| C103 | 7.88 | 7.74 | 0.14 | 7.88 | 6.659 | 1.221 |
| C100 | 7.92 | 8.022 | -0.102 | 7.92 | 7.808 | 0.112 |
| C78 | 8.6 | 8.596 | 0.004 | 8.6 | 8.673 | -0.073 |
| C79 | 9.35 | 9.123 | 0.227 | 9.35 | 9.132 | 0.218 |
| C77 | 9.95 | 9.02 | 0.93 | 9.95 | 9.152 | 0.798 |
| C81 | 10.04 | 9.695 | 0.345 | 10.04 | 9.277 | 0.763 |

Test set data are shown in blue.

**Table S7.** $\lambda$ parameters to gradually change the ligand interaction from state-A to state-B.

| λ | Parameter id | | | | | | | | | | | |
|---|---|---|---|---|---|---|---|---|---|---|---|---|
| | 1 | 2 | 3 | 4 | 5 | 6 | 7 | 8 | 9 | 10 | 11 | 12 |
| lambljA | 1.0 | 0.909 | 0.818 | 0.727 | 0.636 | 0.545 | 0.455 | 0.364 | 0.273 | 0.182 | 0.091 | 0.0 |
| lambljB | 0.0 | 0.091 | 0.182 | 0.273 | 0.364 | 0.455 | 0.545 | 0.636 | 0.727 | 0.818 | 0.909 | 1.0 |
| lambelA | 1.0 | 0.909 | 0.818 | 0.727 | 0.636 | 0.545 | 0.455 | 0.364 | 0.273 | 0.182 | 0.091 | 0.0 |
| lambelB | 0.0 | 0.091 | 0.182 | 0.273 | 0.364 | 0.455 | 0.545 | 0.636 | 0.727 | 0.818 | 0.909 | 1.0 |
| lambbondA | 1.0 | 0.909 | 0.818 | 0.727 | 0.636 | 0.545 | 0.455 | 0.364 | 0.273 | 0.182 | 0.091 | 0.0 |
| lambbondB | 0.0 | 0.091 | 0.182 | 0.273 | 0.364 | 0.455 | 0.545 | 0.636 | 0.727 | 0.818 | 0.909 | 1.0 |

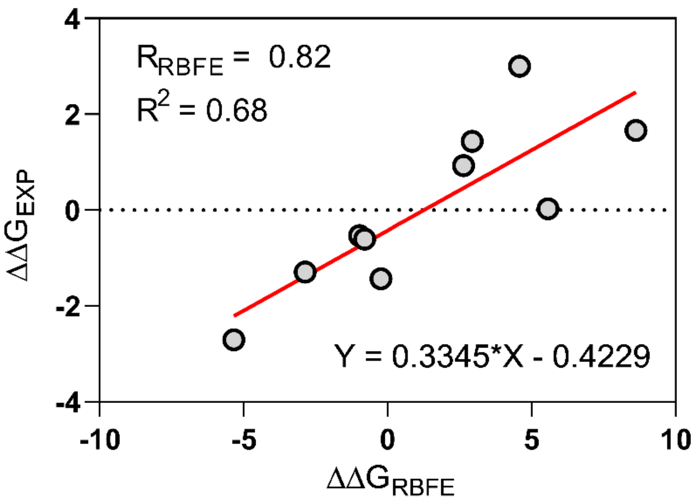


**Figure S2.** Correlation plot between experimental and computed relative binding free energies.
